# Targeting BAP1 with small compound inhibitor for colon cancer treatment

**DOI:** 10.1038/s41598-023-29017-w

**Published:** 2023-02-08

**Authors:** Minhwa Kang, Seul Gi Park, Shin-Ai Lee, Soyi Kim, Daye Lee, Mukesh Eknath Shirbhate, So-Yeon Youn, Kwan Mook Kim, Sun-Shin Cha, Jongbum Kwon

**Affiliations:** 1 Department of Life Science, Ewha Womans University, 52 Ewhayeodae-gil, Seodaemun-gu, Seoul, 03760 Korea; 2 Department of Chemistry and Nanoscience, Ewha Womans University, 52 Ewhayeodae-gil, Seodaemun-gu, Seoul, 03760 Korea; 3 Present Address: Laboratory of Genitourinary Cancer Pathogenesis, Center for Cancer Research, National Cancer Institute, Building 37, Room 1068, MD 20892-4263 Bethesda, USA

**Keywords:** Biochemistry, Cancer, Cell biology, Chemical biology, Computational biology and bioinformatics, Drug discovery, Molecular biology, Gastroenterology, Molecular medicine, Oncology, Chemistry

## Abstract

BRCA1-associated protein-1 (BAP1) is a ubiquitin C-terminal hydrolase domain-containing deubiquitinase. The gene encoding BAP1 is mutated in various human cancers, including mesothelioma, uveal melanoma and renal cell carcinoma. BAP1 plays roles in many cancer-related cellular functions, including cell proliferation, cell death, and nuclear processes crucial for genome stability, such as DNA repair and replication. While these findings suggest that BAP1 functions as a tumor suppressor, recent data also suggest that BAP1 might play tumor-promoting roles in certain cancers, such as breast cancer and hematopoietic malignancies. Here, we show that BAP1 is upregulated in colon cancer cells and tissues and that BAP1 depletion reduces colon cancer cell proliferation and tumor growth. BAP1 contributes to colon cancer cell proliferation by accelerating DNA replication and suppressing replication stress and concomitant apoptosis. A recently identified BAP1 inhibitor, TG2-179-1, which seems to covalently bind to the active site of BAP1, exhibits potent cytotoxic activity against colon cancer cells, with half-maximal inhibitory concentrations of less than 10 μM, and inhibits colon tumor growth. TG2-179-1 exerts cytotoxic activity by targeting BAP1, leading to defective replication and increased apoptosis. This work therefore shows that BAP1 acts oncogenically in colon cancer and is a potential therapeutic target for this cancer. Our work also suggests that TG2-179-1 can be developed as a potential therapeutic agent for colon cancer.

## Introduction

BRCA1-associated protein-1 (BAP1) is a ubiquitin C-terminal hydrolase (UCH) domain-containing deubiquitinase (DUB) that functions in both the nucleus and cytoplasm^1–4^. BAP1 plays a crucial role in various biological processes via interaction with and deubiquitination of histone H2A monoubiquitination at Lys119 (H2A-K119-Ub) and multiple nonhistone substrates. For instance, nuclear BAP1, in cooperation with other factors, acts as both a transcriptional coactivator and transcriptional corepressor to regulate target genes involved in cell cycle progression, cell proliferation, cell death, and metabolic processes^5–10^. BAP1 also promotes apoptosis by direct action at the endoplasmic reticulum, where it stabilizes a factor via deubiquitination to stimulate the physiological release of calcium ions into the cytoplasm and mitochondria^7,11^. In addition, the results of studies using gene targeting approaches suggest that BAP1 functions as a critical regulator of embryonic development and cell differentiation^12–15^.

BAP1 directly participates in chromosomal processes such as DNA repair and replication. BAP1 is recruited to sites of DNA double-strand breaks (DSBs), where it facilitates the assembly of repair proteins at DNA damage lesions and promotes DSB repair^16–18^. A recent work also documented the role of BAP1 in nucleotide excision repair. The study showed that BAP1 is recruited to sites of UV-induced DNA damage via H2A-K119-Ub and PARP1 to promote DNA repair and that both DUB activity and PARP1-mediated poly(ADP-ribosyl)ation are critical for this function of BAP1^19^. During normal DNA synthesis, BAP1 stabilizes the chromatin remodeling factor INO80 via deubiquitination and recruits it to replication forks via H2A-K119-Ub to stimulate fork progression^20,21^. BAP1 also promotes the assembly of Rad51 foci to facilitate DNA repair and replication fork restart during replication stress^22^. Consistent with these functions, both BAP1-deficient cells and cells with transient depletion of BAP1 exhibit chromosomal abnormalities and aneuploidy^16,20,23^, emphasizing the importance of BAP1 in the maintenance of genome integrity.

Initial studies showed that *BAP1* is deleted or mutated in various human cancer cells and that reexpression of BAP1 in H226 human mesothelioma cells, which lack intrinsic BAP1 expression, reverses their tumorigenicity^24,25^. A series of subsequent studies revealed inactivating mutations in *BAP1* in diverse human cancers, including mesothelioma, uveal melanoma, cutaneous melanoma, and renal cell carcinoma^26–31^. During their lifetime, all carriers of inherited heterozygous *BAP1*-inactivating mutations develop at least one cancer and often multiple cancers, mostly malignant mesothelioma and uveal melanoma (called BAP1 cancer syndrome). Cells from carriers with pathogenic *BAP1* mutations are prone to malignant transformation, and acquired biallelic *BAP1* mutations are common in human cancers^2,31,32^. Gene targeting studies have reported that *BAP1* disruption in hematopoietic lineage cells of adult mice leads to the development of myeloid neoplasia with features of human chronic myelomonocytic leukemia. In addition, mice carrying heterozygous germline *BAP1* mutations develop various spontaneous tumors and are predisposed to the development of malignant mesothelioma after exposure to carcinogenic fibers (asbestos)^12,13,33^. These results, together with cancer-related cellular activities of BAP1, such as those supporting cell proliferation and genome stability, suggest that BAP1 functions as a tumor suppressor.

Recent studies suggest that BAP1 might also play tumor-promoting roles in certain types of cancer. For instance, BAP1 deubiquitinates and stabilizes the transcription factor KLF5, which is highly expressed in breast cancer and a potent biomarker for unfavorable prognosis in breast cancer patients, and promotes breast cancer cell proliferation, survival, and migration as well as tumor growth^34^. A truncation mutation of BAP1-associated protein ASXL1, frequently found in hematopoietic malignancies, confers enhanced activity of the ASXL1–BAP1 complex and acts as a gain-of-function mutation to promote myeloid leukemogenesis^35,36^. In the present study, we investigated whether BAP1 functions oncogenically in colon cancer. We showed that BAP1 is upregulated in colon cancer cells and tissues and that BAP1 depletion reduces colon cancer cell proliferation and tumor growth. We also suggest that a recently identified BAP1 inhibitor can be developed as a potential therapeutic agent for colon cancer.

## Materials and methods

### Cells and antibodies

HCT116, HCT15, HT29, SW48, SW480, RKO, DLD-1, LoVo, CCD-112CoN (CRL-1542), MSTO-211H, H226, 786-O and Umrc-6 cells were purchased from the American Type Culture Collection (ATCC; Manassas, VA), and CCD-18Co cells were purchased from the Korean Cell Line Bank (KCLB; Seoul, Korea). Cells were cultured in the following base media supplemented with 10% fetal bovine serum (FBS; Gibco, Carlsbad, CA), 100 U/ml penicillin, and 100 μg/ml streptomycin: RPMI 1640 medium for HCT15, SW48, SW480, DLD-1, LoVo, MSTO-211H, H226, and 786-O cells; McCoy’s 5A (modified) medium for HCT116 and HT29 cells; Dulbecco’s modified Eagle’s medium for CCD-112CoN, CCD-18Co and Umrc-6 cells; and Eagle’s minimum essential medium for RKO cells. All colon cancer cells were authenticated by DNA fingerprinting using short tandem repeat (STR) markers every 50 passages and were tested for the absence of *Mycoplasma* contamination using an e-Myco VALiD Mycoplasma PCR Detection Kit (Intron Biotechnology). All cells were maintained at 37 °C in a humidified incubator with 5% CO_2_. The antibodies used in this work and their sources are as follows: anti-BAP1 (sc-28383), anti-α-Tubulin (sc-8035) and anti-Actin (sc-8432) purchased from Santa Cruz; anti-Axin1 (C76H11) and anti-β-Catenin (6B3) purchased from Cell Signaling Technology; anti-H2A (07-146) and anti-γ-H2A (05-636) purchased from Millipore; and anti-GAPDH (LF-PA0212) purchased from AbFrontier.

### Tissue microarray analysis

Five-micrometer-thick paraffin-embedded frozen tissue sections mounted on slides (US Biomax, BC051110c) were deparaffinized in xylene and rehydrated using decreasing concentrations of ethanol. The tissue sections were then incubated in sodium citrate buffer (10 mM sodium citrate, 0.05% Tween 20 (pH 6.0)) at 95 °C for 30 min for antigen retrieval and treated with 3% hydrogen peroxide for 5 min to block endogenous peroxidase activity. For blocking, the slides were incubated in PBS containing 2% bovine serum albumin (BSA) at room temperature (RT) for 1 h. Primary anti-BAP1 antibodies diluted in 2% blocking serum (1:200) were added to the tissue sections and incubated overnight at 4 °C. After several washes with 0.01% Triton X-100 in PBS, the tissue sections were sequentially incubated with prediluted biotinylated goat anti-rabbit IgG (Vector Laboratories) and VECTASTAIN ABC Reagent (Vector Laboratories). Immunoreactive cells were visualized by the addition of DAB chromogen (Vector Laboratories) and hematoxylin counterstain. Images were acquired using an Olympus BX 51 light microscope.

### Immunoblot analysis

Immunoblot analysis was performed according to a standard method. In brief, cells were washed with PBS and lysed in radioimmunoprecipitation assay (RIPA) buffer (50 mM Tris–Cl (pH 8.0), 150 mM NaCl, 0.5% sodium deoxycholate, 0.1% sodium dodecyl sulfate (SDS), 1% NP-40, 1 mM dithiothreitol (DTT), 0.5 mM phenylmethylsulfonyl fluoride (PMSF), 5 μg/ml aprotinin, 5 μg/ml leupeptin, 5 μg/ml pepstatin A, and 10 mM NaF) at 4 °C for 30 min. Protein concentrations were measured by a bicinchoninic acid (BCA) assay. After separation on an SDS–polyacrylamide gel, proteins were transferred onto a nitrocellulose membrane (GE Healthcare Life Sciences) using standard transfer buffer (25 mM Tris base, 192 mM glycine, and 20% methanol) for nonhistone proteins and N-cyclohexyl-3-aminopropanesulfonic acid (CAPS) buffer (25 mM CAPS and 20% methanol) for histones. Signals were detected by enhanced chemiluminescence (ECL; Young-In Frontier).

### siRNAs and transfection

Transfection with synthetic siRNAs was performed using Lipofectamine RNAiMAX (Invitrogen). The sequences of the siRNAs used were as follows: BAP1 siRNA (si-BAP1)-1, 5'-cuccaucagaccaauccaa (dTdT)-3'; si-BAP1-2, 5'-gugaucuggguccugucau(dTdT)-3'; si-BAP1-3, 5'-cuguucaguggccugugugaa (dTdT)-3'.

### Colony formation assay

Cells (1 × 10^3^ cells/dish) were seeded in 35-mm dishes in triplicate and incubated for 10–14 days. After the colonies were stained with 0.5% crystal violet, 10% acetic acid was used to solubilize the remaining crystal violet before measurement of the absorbance at 590 nm using a SpectraMax i3X plate reader (I3X-SC-ACAD, Molecular Devices).

### MTS assay

A total of 1 × 10^3^ cells/well in 100 μl of medium were seeded in a 96-well plate and treated with TG2-179-1. After 72 or 120 h, the cells were subjected to an MTS assay according to the protocols of the CellTiter 96 AQueous One Solution Cell Proliferation Assay (Promega). The absorbance at 490 nm was measured using a SpectraMax i3X microplate reader (I3X-SC-ACAD, Molecular Devices).

### Apoptosis assay

Annexin-V/PI double staining was performed using a FITC Annexin V Apoptosis Detection Kit I (556547, BD Bioscience) according to the manufacturer’s protocol. After two washes with PBS, 2 × 10^3^ cells were resuspended in 1 ml of binding buffer. One hundred microliters of the cell suspension (1 × 10^5^ cells) was mixed with 5 μl of Annexin V-FITC and 5 μl of propidium iodide (PI; Invitrogen). After gentle vortexing, the cells were incubated at RT for 20 min in the dark before being subjected to flow cytometric analysis using a FACSCalibur instrument (BD Biosciences).

### Lentivirus production

The lentiviral vectors expressing Flag-BAP1 and Flag-BAP1-C91S^20^ were transfected into 293 T cells using FuGENE HD transfection reagent (Promega). After 48 h, the lentivirus-containing medium was harvested and filtered through a 0.45 μm syringe filter to remove residual 293 T cells. The filtered lentivirus-containing medium was used for cell infection.

### DNA fiber assay

The DNA fiber assay was performed as previously described^20^. Cells were incubated sequentially with 100 µM 5-iodo-2′-deoxyuridine (IdU) and 5-chloro-2′-deoxyuridine (CldU) for 20 min each, harvested by trypsinization and resuspended in PBS. Approximately 200–400 cells/slide were plated on microscope slides (Marienfeld) and air dried for 7–10 min, and 10 μl of spreading buffer (0.5% SDS, 200 mM Tris–Cl (pH 7.5), and 50 mM EDTA) was then added. After 10 min of incubation, the slides were tilted slowly for 2–4 h to allow the DNA fibers to extend. After fixation with acetic acid:methanol (1:3) for 2 min, the cells on the slides were washed with PBS. The slides were stored at − 20 °C overnight and were then treated with 500 μl of 2.5 M HCl for 30 min to denature the extended DNA fibers, washed with PBS, and incubated in 2% BSA for 40 min. The slides were incubated first with primary antibodies (mouse anti-BrdU for detection of IdU; rat anti-BrdU for detection of CldU) at RT for 1 h and then with stringency buffer (10 mM Tris–Cl (pH 7.5), 400 mM NaCl, 0.2% Tween 20, and 0.2% NP-40) at RT for 10 min to remove nonspecifically bound antibodies. After three washes with PBS, the slides were incubated with secondary antibodies (Alexa Fluor 568 rabbit anti-mouse IgG (Invitrogen); Alexa Fluor 488 chicken anti-rat IgG (Invitrogen)) at RT for 30 min followed by another secondary antibody (Alexa Fluor 568 goat anti-rabbit IgG (Invitrogen); Alexa Fluor 488 goat anti-chicken IgG (Invitrogen)). The slides were then blocked with 2% BSA for 15 min. After washing, the slides were mounted using Vectashield mounting medium and imaged using a Carl Zeiss LSM 880 confocal microscope.

### Xenograft experiments

After transfection with control or BAP1 siRNAs, HCT116 cells (3 × 10^6^) were suspended in 100 μl of PBS containing 20% Matrigel (Corning) and injected subcutaneously into the backs of 5-week-old male BALB/c-nude mice (Orient Bio Inc, Korea). Beginning 1 week after inoculation, tumor sizes were measured with a caliper twice weekly, and tumor volumes were calculated using the formula V = 1/2 × L × W^2^. For TG2-179-1 treatment, HCT116 cells (3 × 10^6^) suspended in 100 μl of PBS containing 20% Matrigel (Corning) were injected subcutaneously into the backs of nude mice. Beginning 1 week after inoculation, DMSO (vehicle) and TG2-179-1 (10 mg/kg or 30 mg/kg) dissolved in PBS containing 20% Kolliphor EL (Sigma) were injected intraperitoneally into the mice (six mice per condition) three times weekly for three weeks. Tumor sizes were measured as described above. All mouse experiments were performed in compliance with the ARRIVE guidelines and approved by the Institutional Animal Care and Use Committee (IACUC) of Ewha Womans University, South Korea.

### DUB activity assay with Ub-AMC

The DUB activity of BAP1 was measured using the ubiquitin-7-amido-4-methylcoumarin (Ub-AMC) DUB assay system (Boston Biochem) as previously described^19^. In brief, Ub-AMC was incubated with His-Flag-BAP1 or His-Flag-BAP1-C91S in a black 96-well plate at RT, and fluorescence signals were measured every 15 s using a SpectraMax i3X microplate reader (I3X-SC-ACAD, Molecular Devices) at an excitation wavelength of 350 nm and an emission wavelength of 455 nm. For the DUB assay for in vitro immunoprecipitated BAP1, total reaction volumes of 20 μl containing 200 ng of Flag-His-BAP1 and TG2-179-1 (1 mM) in the reaction buffer (20 mM HEPES (pH 7.5), 50 mM NaCl, 20 mM MgCl_2_, and 1 mM DTT) were incubated at RT for 1 h. These reactions were mixed with 800 μl of a buffer containing 1% NP-40 and incubated with 10 μl of anti-Flag M2 affinity gel (Sigma) overnight at 4 °C. The beads were washed three times with buffer containing 1% NP-40 and were then resuspended in DUB reaction buffer containing Ub-AMC before measurement of fluorescence signals as described above.

### DUB assay with H2A-Ub mononucleosomes

The DUB assay using H2A-Ub nucleosome substrates was performed as previously described^19^. In brief, TG2-179-1 was mixed with His-Flag-BAP1 or His-Flag-BAP1-C91S, and the reactions were incubated with H2A-Ub mononucleosomes (EpiCypher) and ASXL1^DEU^ in DUB buffer (50 mM Tris–Cl (pH 7.5), 50 mM NaCl_2_, 1 mM MgCl_2_, and 1 mM DTT) at RT for 90 min. Reactions were terminated by the addition of equal volumes of SDS sample loading buffer and boiled for 5 min before being subjected to SDS–PAGE and immunoblot analysis.

### Preparation of recombinant proteins

His-Flag-BAP1, His-Flag-BAP1-C91S and His-ASXL1^DEU^ were expressed in and purified from *E. coli* as previously described^19^.

### In silico molecular docking studies

TG2-179-1 was docked into the active site of the BAP1 structure by using AutoDock Vina^37^. The BAP1 structure was generated with SWISS-MODEL, a fully automated homology modeling server, with the crystal structure of UCH (PDB code: 4WLR) as the template. The two-dimensional structure of TG2-179-1 was generated in ChemDraw 20.1.1 and was then converted to the three-dimensional structure in Avogadro. Polar hydrogen atoms were added to the BAP1 structure, and side chain conformational changes were allowed for the catalytic triad (Cys91, His169, and Asp184) during docking.

### TG2-179-1

The structural information for TG2-179-1 was obtained from the PubChem database (CID: 24178338, https://pubchem.ncbi.nlm.nih.gov/). TG2-179-1 was synthesized as detailed in the Supplementary Methods S1, and the purity was estimated to be > 99.5%. The inhibitor was dissolved in DMSO, stored in aliquots at − 20 °C, and thawed immediately before use for experiments.

### Statistical analysis

All experimental data are expressed as the mean ± s.d. values wherever applicable. The significance of differences between measurements was evaluated by Student’s *t* test using Microsoft Excel. On the graphs, *p* values are indicated by asterisks as follows: *, *p* < 0.05; **, *p* < 0.01; ***, *p* < 0.001. A *p* value > 0.05 was considered nonsignificant (*ns*) unless otherwise indicated. Half-maximal inhibitory concentration (IC_50_) values were calculated with GraphPad Prism software.

All methods were carried out in accordance with relevant guidelines and regulations. Original gels/blots are presented in Supplementary material S1. All blots except the one in Fig. 5D and a few in Figs. 4F and 8D were cut, mostly horizontally, prior to hybridization with antibodies.

## Results

### BAP1 is upregulated in colon cancer

**Figure 1:**
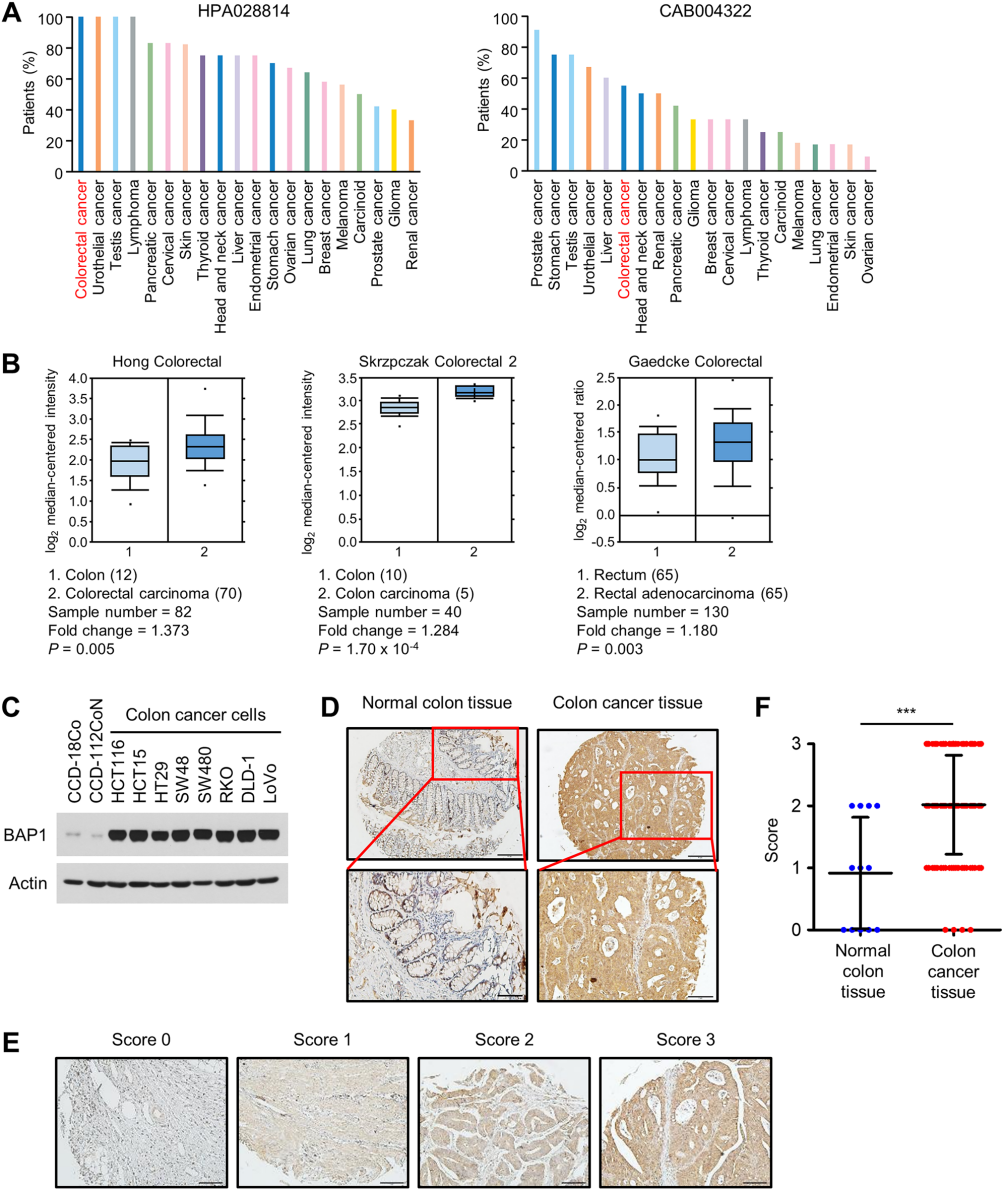
BAP1 is upregulated in colon cancer. (**A**) BAP1 expression was determined in various cancer types by immunohistochemistry using anti-BAP1 antibodies from two different sources, i.e., HPA028814 and CAB004322. For each cancer type, the color-coded bars indicate the percentages of patients with high and medium protein expression levels. For colorectal cancer, 12 of 12 patients (with HPA028814) and 6 of 12 patients (with CAB004322) showed high/medium levels of BAP1 expression. The graph was taken from the Human Protein Atlas database (https://www.proteinatlas.org/). (**B**) Results of differential analysis of BAP1 mRNA expression in normal colorectal and colorectal cancer tissues using the Oncomine database (https://www.oncom ine.org/resource). Only output results with *p* < 0.05 were retained. The number in parentheses is the sample number. (**C**) Results of immunoblot analysis of BAP1 in various normal colon cells and colon cancer cells. Actin was used as the loading control. (**D**) Representative images of immunohistochemical staining for BAP1 using a tissue microarray (US Biomax, BC051110c, Supplementary Fig. S1A,B) containing normal colon tissues (*n* = 12) and colon cancer tissues (*n* = 108). Scale bars: 200 µm (top), 100 µm (bottom). More staining images are provided in Supplementary Fig. S1C. (**E**) The results of immunohistochemical scoring BAP1 expression. The expression level of BAP1 was scored on a scale from 0 to 3. Scores of 0 and 1 were considered negative and scores of 2 and 3 were considered positive for BAP1 expression. Scale bar, 100 µm. (**F**) The BAP1 expression score for 12 normal colon tissues and 108 colon cancer tissues was depicted as a scatter plot. The lines indicate the mean ± s.d. values.

To determine the status of BAP1 expression in colon cancer, we searched several online databases. Immunohistochemical staining data from the Human Protein Atlas showed that 100% and 55% of examined colon cancer patients exhibited high and medium BAP1 expression levels, respectively, depending on the source of the antibodies used (Fig. 1A). Differential analysis of normal and cancer tissues in 11 colorectal datasets comprising 1,249 samples revealed that BAP1 mRNA expression was upregulated in colorectal cancer tissues compared to normal colorectal tissues (Fig. 1B). Then, we compared BAP1 expression between normal colon cells and colon cancer cells by immunoblot analysis. All eight colon cancer cell lines analyzed exhibited markedly higher BAP1 levels than the two analyzed normal colon cell lines (Fig. 1C). In addition, we analyzed BAP1 expression in colon adenocarcinoma and normal colon tissues using a tissue microarray (Supplementary Fig. S1A,B). We scored BAP1 staining on a scale of 0 to 3 and arbitrarily designated scores of 0 and 1 as BAP1-negative and scores of 2 and 3 as BAP1-positive. Approximately 77% of the colon cancer tissues (83 of 108) but only 33% of the normal colon tissues (4 of 12) were scored as BAP1-positive (Fig. 1D–F, Supplementary Fig. S1C). Collectively, these data indicate that BAP1 is upregulated in colon cancer.

### BAP1 is critical for the viability of various colon cancer cells

**Figure 2:**
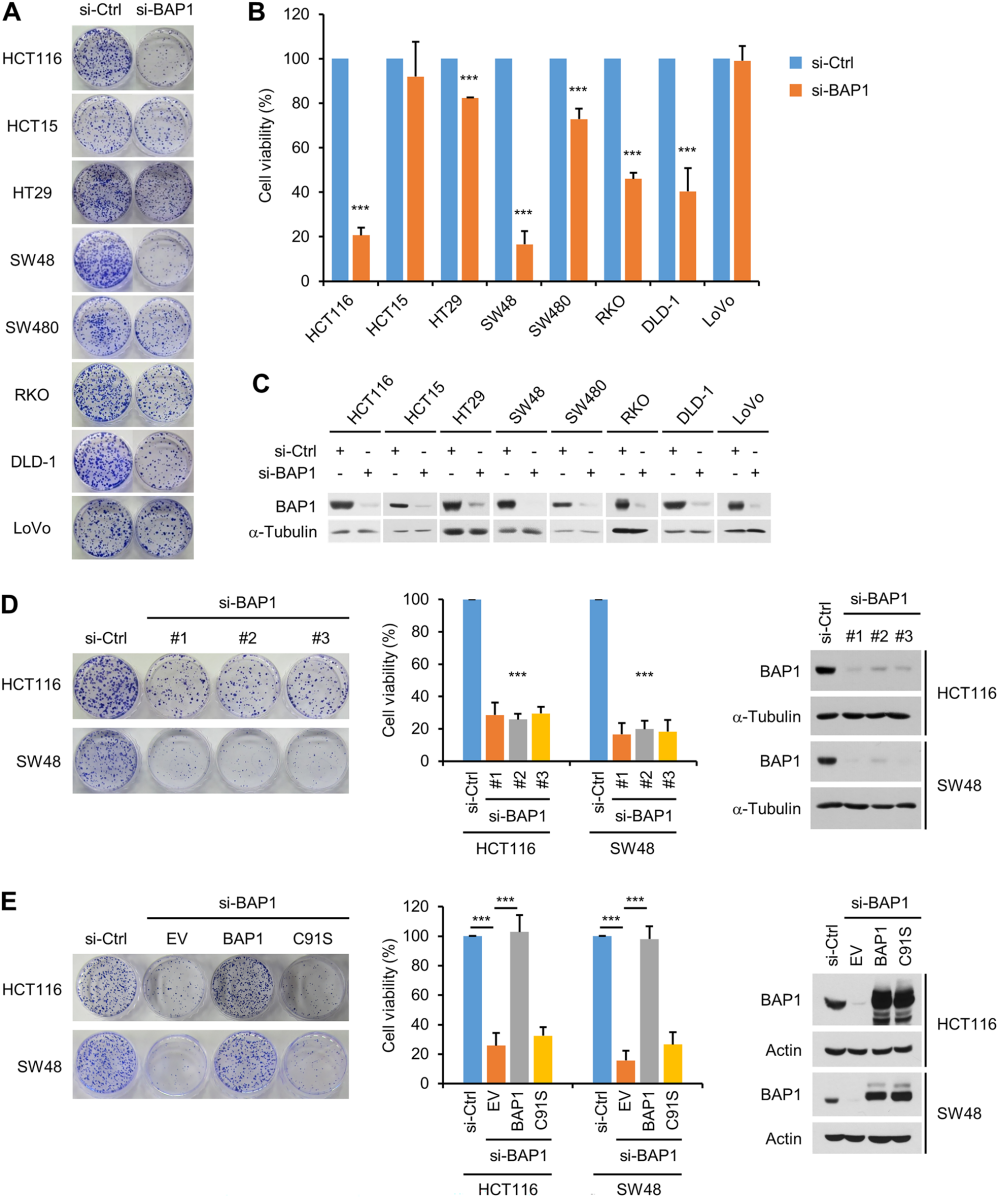
BAP1 is critical for the viability of various colon cancer cells. (**A**) Various colon cancer cells were transfected with control or BAP1 siRNAs and subjected to a colony formation assay. Representative results are shown. (**B**) The results of the colony formation assay are depicted graphically. *n* = 3 (each performed in triplicate, applicable to all colony formation assays in this paper); error bars, mean ± s.d. values. (**C**) Results of immunoblot analysis of BAP1 knockdown. α-Tubulin was used as the loading control. (**D**) (Left) Representative results of the colony formation assay in HCT116 and SW48 cells after BAP1 knockdown by three siRNAs targeting different sequences. (Middle) The results of the colony formation assay are depicted graphically. *n* = 3; error bars, mean ± s.d. values. (Right) Results of immunoblot analysis of BAP1 knockdown. (**E**) After HCT116 and SW48 cells were transfected with control or BAP1 siRNAs, the si-BAP1-transfected cells were transduced with an empty lentiviral vector (EV) or lentiviral vectors expressing wild-type or C91S mutant forms of Flag-BAP1, which were then subjected to a colony formation assay. (Left) Representative results of the colony formation assay. (Middle) Summary of the results of the colony formation assay. *n* = 3; error bars, mean ± s.d. values. (Right) Results of immunoblot analysis.

The upregulation of BAP1 in colon cancer led us to investigate whether BAP1 regulates the viability of colon cancer cells. We depleted BAP1 in the aforementioned eight colon cancer cell lines by a specific siRNA and evaluated their viability by a colony formation assay. Six of the eight cell lines (HCT116, HT29, SW48, SW480, RKO and DLD-1) showed decreased viability after BAP1 depletion, with the remaining two (HCT15 and LoVo) showing little effect. Among the six cell lines with decreased viability, HCT116 and SW48 cells were the most markedly affected by BAP1 depletion, with a decrease in viability of more than 80% (Fig. 2A–C). We confirmed the viability decrease in these two cell lines after BAP1 depletion by transfection of two more siRNAs that targeted different sequences (Fig. 2D). Notably, complementation by BAP1 but not by the C91S catalytic mutant rescued the reduced viability of BAP1-depleted HCT116 and SW48 cells (Fig. 2E). Therefore, the activity of BAP1 to support the viability of these colon cancer cells is specific and dependent on its DUB activity.

### BAP1 depletion increases apoptosis in colon cancer cells

**Figure 3:**
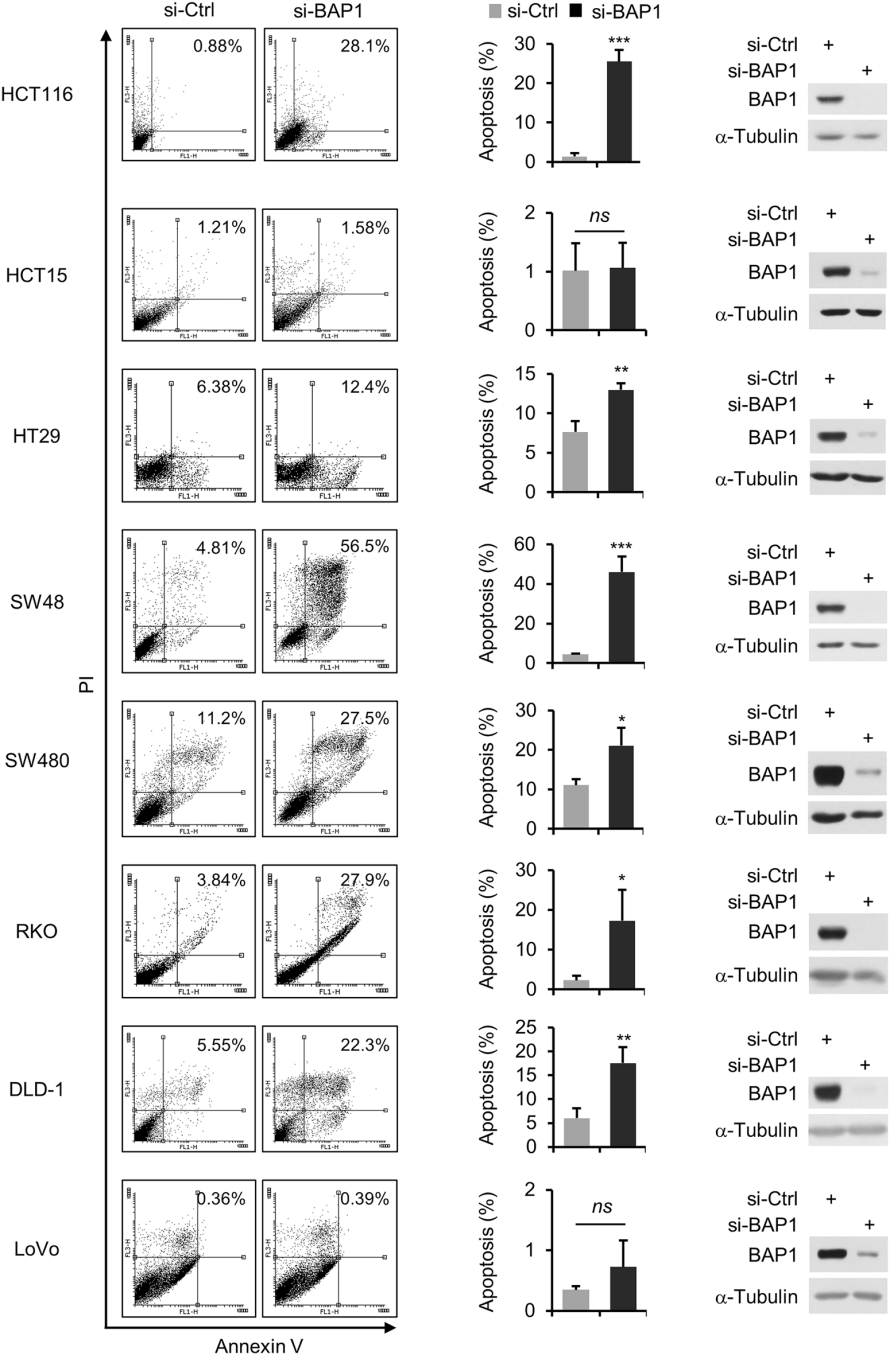
BAP1 depletion increases apoptosis in colon cancer cells. Results of the apoptosis assay in colon cancer cells. After transfection with control or BAP1 siRNAs, cells were subjected to Annexin V/PI double staining followed by FACS analysis. Representative results are shown (left), along with summary graphs (middle, *n* = 3; error bars, mean ± s.d. values) and the results of immunoblot analysis for BAP1 knockdown (right).

To confirm the results of the colony formation assay and determine the mechanisms by which BAP1 activity supports colon cancer cell viability, we analyzed apoptosis by Annexin-V/PI double staining. BAP1 depletion increased apoptosis in HCT116, HT29, SW48, SW480, RKO and DLD-1 cells, with little effect on HCT15 and LoVo cells (Fig. 3). Among the six affected cell lines, HCT116, SW48 and RKO cells exhibited the most marked increase in apoptosis, with increases of approximately 19-, 11- and eightfold, respectively, while the other three showed increases ranging from two- to threefold (Fig. 3). Unlike the increase in apoptosis, the increase in necrosis after BAP1 depletion was nonsignificant. These results indicate that the reduction in colon cancer cell viability induced by BAP1 depletion was attributed mainly to increased apoptosis.

### BAP1 depletion induces replication defects in colon cancer cells and inhibits colon tumor growth

**Figure 4:**
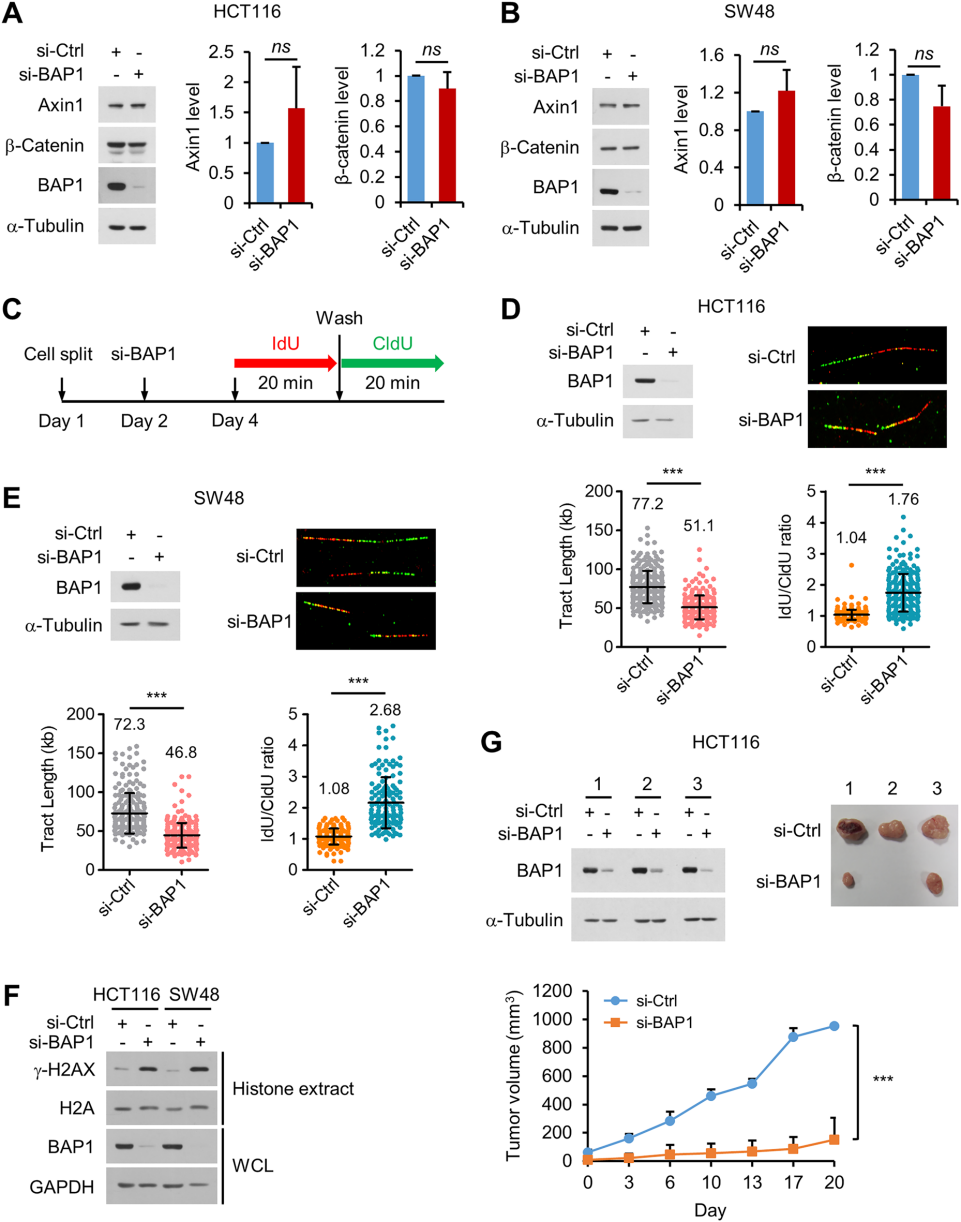
BAP1 depletion induces replication defects in colon cancer cells and inhibits colon tumor growth. (**A**,**B**) After transfection with control or BAP1 siRNAs, Axin1 and β-catenin expression in HCT116 (**A**) and SW48 cells (**B**) was analyzed by immunoblotting. *n* = 3; error bars, mean ± s.d. values. (**C**) Experimental scheme. (**D**,**E**) After transfection with control or BAP1 siRNAs, HCT116 (**D**) and SW48 cells (**E**) were subjected to a DNA fiber assay. (Upper left) Immunoblot analysis of BAP1 knockdown. (Upper right) Representative images of DNA fibers. (Lower left) Summary of the IdU and CldU track lengths. Fiber counts for the scatter plot: si-Ctrl = 265, si-BAP1 = 228 for HCT116; si-Ctrl = 198, si-BAP1 = 248 for SW48. (Lower right) Summary of the IdU/CldU ratio. Fiber counts for the scatter plot: si-Ctrl = 229, si-BAP1 = 250 for HCT116; si-Ctrl = 168, si-BAP1 = 152 for SW48. (**F**) After BAP1 knockdown, cells were divided into two groups for preparation of whole-cell lysates (WCLs) and histone extracts for immunoblotting. Representative data from three similar results are shown. (**G**) Results of the xenograft experiment showing that BAP1 knockdown inhibited the tumorigenic growth of HCT116 cells. (Upper left) Results of immunoblot analysis of BAP1 knockdown in three separate experiments. (Upper right) Images of the tumors excised from mice. (Bottom) Summary graph. *n* = 3; error bars, mean ± s.d. values.

We further investigated the mechanisms by which BAP1 supports the viability of colon cancer cells by focusing on HCT116 and SW48 cells, which exhibited the highest sensitivity to BAP1 deficiency among the eight cell lines analyzed. Adenomatous polyposis coli (Apc) plays a crucial role in colon cancer cell proliferation and tumorigenesis. Apc, in cooperation with other factors, such as the Axin scaffold protein, triggers proteolytic cleavage of β-catenin via phosphorylation-induced ubiquitination, and defects in the function of Apc lead to nuclear accumulation of β-catenin, resulting in the activation of target genes that stimulate cell cycle progression^38,39^. While HCT116, SW48 and RKO cells harbor wild-type Apc, the remaining five tested cell lines carry inactivating mutations in Apc (Supplementary Fig. S2), indicating that the requirement of BAP1 for the viability of colon cancer cells is not clearly correlated with the activity of Apc. In addition, BAP1 depletion did not affect the levels of Axin and β-catenin in HCT116 and SW48 cells (Fig. 4A,B). Therefore, it is unlikely that BAP1 contributes to the survival and proliferation of colon cancer cells via the Apc/β-catenin pathway.

BAP1 functions in both normal DNA replication and replication stress recovery. We therefore investigated whether BAP1 affects these processes in HCT116 and SW48 cells. After BAP1 depletion, we treated HCT116 cells sequentially with IdU and CldU to label DNA. We then allowed the DNA to extend and analyzed incorporation of the thymidine analogs into the DNA by immunofluorescence microscopy (Fig. 4C). BAP1 depletion reduced the speed of replication fork progression in the cells, as indicated by the decreases in the IdU and CldU track lengths (Fig. 4D). To identify stalled and collapsed forks, we analyzed the symmetry between the IdU and CldU track lengths in the labeled DNA fibers. The average IdU/CldU ratio was approximately 1, which is also the theoretical expected value, but increased to 1.76 after BAP1 depletion (Fig. 4D), indicating that BAP1 deficiency resulted in fork stalling and collapse. We repeated these experiments with SW48 cells and observed similar results: BAP1 depletion reduced the speed of replication fork progression and resulted in fork stalling and collapse (Fig. 4E). Consistent with these results, BAP1 depletion increased the level of γ-H2AX, the cellular marker of stalled and collapsed forks, in both HCT116 and SW48 cells (Fig. 4F). These data show that BAP1 is critical for ensuring efficient fork progression and suppressing replication stress in colon cancer cells.

Given the critical role of BAP1 in DNA replication and the survival of colon cancer cells, we performed a xenograft experiment in mice to determine whether BAP1 is required for the growth of colon cancer cells into tumors. We inoculated BAP1-depleted and control HCT116 cells into athymic nude mice and allowed the cells to grow for up to 20 days. The growth of the BAP1-depleted cells into tumors was greatly suppressed compared to that of control cells (Fig. 4G), showing that BAP1 is critical for the tumorigenic growth of colon cancer cells.

### TG2-179-1 inhibits the DUB activity of BAP1 by binding to its active site

**Figure 5:**
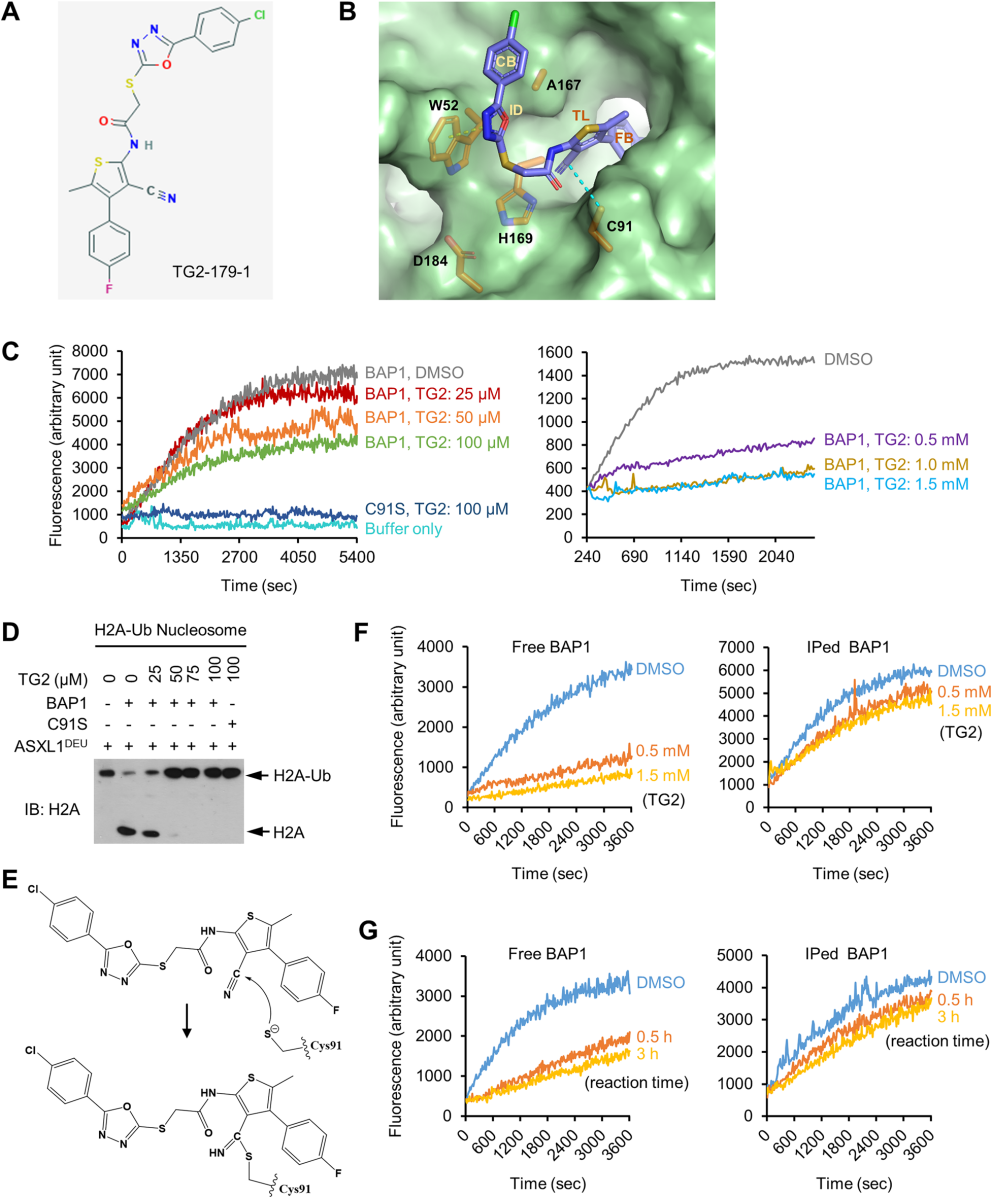
TG2-179-1 inhibits the DUB activity of BAP1 by binding to its active site. (**A**) Structure of TG2-179-1. (**B**) In silico docking model of the BAP1–TG2-179-1 complex. The active site residues interacting with TG2-179-1 (Trp52 and Ala167) and the catalytic triad (Cys91, His169, and Asp184) are shown as orange-colored sticks. The yellow dashed line indicates the $\pi$-$\pi$ stacking between the 2-imidazolidone ring of TG2-179-1 and Trp52 of BAP1, while the cyan dashed line shows the distance between the nitrile carbon atom of TG2-179-1 and the side-chain sulfur atom of Cys91. Chloride, nitrogen, oxygen, and sulfur atoms of TG2-179-1 are colored green, blue, red, and yellow, respectively. CB, ID, TL, and FB stand for chlorobenzene, 2-imidazolidone, thiolane, and fluorobenzene, respectively. (**C**) BAP1 was incubated with increasing concentrations of TG2-179-1 (from 25 μM to 1.5 mM; DMSO as the vehicle control) at RT for 1 h, and the reactions were then subjected to a Ub-AMC DUB assay. BAP1-C91S was included as a control. A representative of several similar results is shown. (**D**) BAP1 was incubated with increasing concentrations of TG2-179-1 and subjected to the H2A-Ub nucleosome DUB assay. BAP1-C91S was included as a control. Representative data from three similar results are shown. (**E**) Proposed mechanism for the inhibition of BAP1 by TG2-179-1. See the text for details. (**F**) After incubation with increasing concentrations of TG2-179-1, BAP1 was immunoprecipitated (IPed) with extensive washing, and the immune complexes were subjected to a Ub-AMC DUB assay. Similar experiments were performed with free BAP1 (no IP) as a control. (**G**) Results of the experiments similar to those in (**F**) but with incubation of BAP1 with TG2-179-1 for increasing reaction times. Representative data from three similar results are shown in (**F**) and (**G**).

A small compound inhibitor of BAP1, termed TG2-179-1, was identified by screening using a high-throughput kinetic BAP1 activity assay. We obtained the structural information of TG2-179-1 from the PubChem database (Fig. 5A) and chemically synthesized this compound. To predict the binding mode of TG2-179-1 to BAP1, we performed in silico molecular docking studies. The active site of BAP1 contains a catalytic triad consisting of Cys91, His169 and Asp184, in which Cys91 acts as a nucleophile^40^. TG2-179-1 is characterized by four rings: chlorobenzene, 2-imidazolidone, thiolane and fluorobenzene. According to the docking model, the fluorobenzene ring and its adjacent thiolane ring are deeply embedded into a pocket at the bottom of the active site (Fig. 5B). The 2-imidazolidone and chlorobenzene rings lean against Trp52 and Ala167 to form a $\pi$-$\pi$ stacking interaction and a hydrophobic contact, respectively (Fig. 5B).

We then determined whether TG2-179-1 inhibits BAP1 using purified recombinant BAP1 proteins and the fluorogenic artificial DUB substrate Ub-AMC as a BAP1 substrate. Incubation of BAP1 with increasing concentrations of TG2-179-1 reduced the emission of fluorescence from Ub-AMC in a dose-dependent manner (Fig. 5C), indicating that TG2-179-1 inhibited the DUB activity of BAP1. The BAP1-C91S catalytic mutant, used as a control, exhibited no activity toward Ub-AMC and was not affected by TG2-179-1 (Fig. 5C). Next, we verified the inhibitory activity of TG2-179-1 against BAP1 using a more physiological substrate, H2A-K119-Ub (hereafter referred to as H2A-Ub), which was assembled into nucleosomes. Increasing concentrations of TG2-179-1 were incubated with H2A-Ub nucleosomes containing the DEUBAD domain of ASXL1 (ASXL1^DEU^), which activates BAP1 by increasing its affinity for the ubiquitin moiety of H2A-Ub^41^. TG2-179-1 inhibited the DUB activity of BAP1 toward nucleosomal H2A-Ub substrates in a dose-dependent manner (Fig. 5D). In contrast, TG2-179-1 had no effect on BAP1-C91S, as expected (Fig. 5D). These data clearly demonstrated that TG2-179-1 inhibits the DUB activity of BAP1.

Analysis of the structural model of the BAP1–TG2-179-1 complex using the CavityPlus server showed that Cys91 was predicted to form a covalent bond with an electron-deficient atom in TG2-179-1^42^. This nitrile carbon atom was sufficiently electrophilic to be attacked by the nucleophilic cysteine^43^. Remarkably, our docking model supported this prediction; in the model, the nitrile carbon atom of TG2-179-1 is located near Cys91, a phenomenon achieved via anchoring of the fluorobenzene and thiolane rings in the BAP1 active site pocket. The distance between the two atoms is ~ 4.1 Å, which is short enough to allow the nucleophilic attack of the nitrile carbon atom by Cys91 (Fig. 5E)^44^. These modeling results suggested that TG2-179-1 is likely a covalent inhibitor of BAP1. To test this hypothesis, we immunoprecipitated TG2-179-1–bound BAP and determined whether BAP1 remained inhibited after extensive washing. Immunoprecipitated BAP1 (in the immune complexes), compared to free BAP1 in the parallel reactions, was only partially inhibited by TG2-179-1 in a manner dependent on the TG2-179-1 concentration (Fig. 5F) and reaction time (Fig. 5G). One feasible explanation for these results is that TG2-179-1 acts as a reversible covalent inhibitor, as previously reported for inhibitors of other proteins^45^.

### TG2-179-1 has potent cytotoxic activity against colon cancer cells

**Figure 6:**
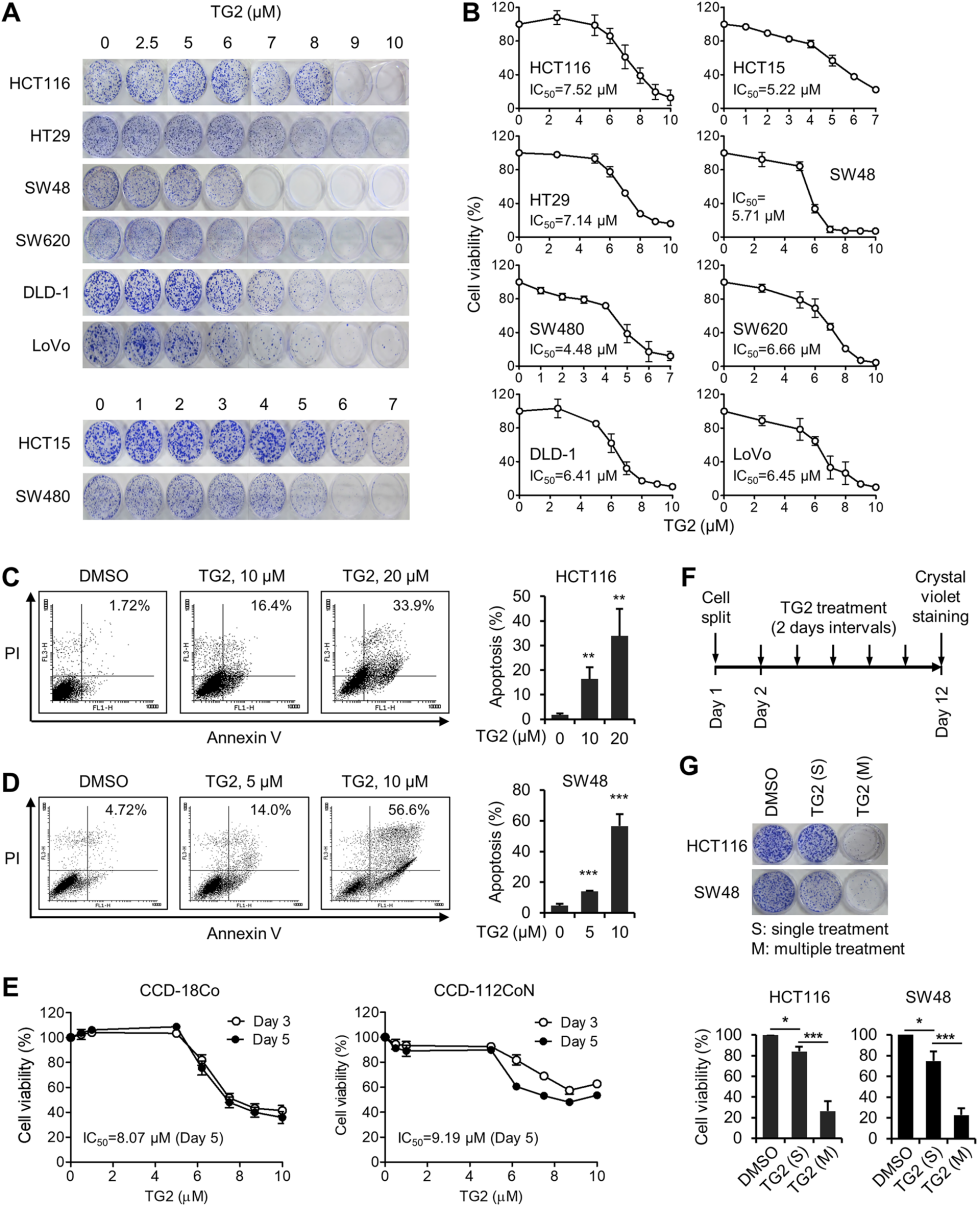
TG2-179-1 has potent cytotoxic activity against colon cancer cells. (**A**) Colon cancer cells were incubated with increasing concentrations of TG2-179-1, and viability was evaluated by a colony formation assay. A representative result for each cell line is shown. (**B**) The results of the colony formation assay are depicted graphically. *n* = 3; error bars, mean ± s.d. values. The IC_50_ values are shown. (**C**,**D**) HCT116 (**C**) and SW48 cells (**D**) were incubated with increasing concentrations of TG2-179-1 for 48 h and subjected to Annexin V/PI double staining followed by FACS analysis. Representative results are shown (left), along with a summary graph (right). *n* = 3; error bars, mean ± s.d. values. (**E**) CCD-18Co and CCD-112CoN normal colon cells were incubated with increasing concentrations of TG2-179-1, and viability was determined on days 3 and 5 by MTS assay. The IC_50_ values for day 5 are shown. (**F**) Experimental scheme. (**G**) HCT116 and SW48 cells were treated with 8 and 5 μM TG2-179-1, respectively, either in a single treatment or multiple treatments every other day with washout, and viability was evaluated by a colony formation assay. Representative data from three similar results are shown, along with a summary graph. *n* = 3; error bars, mean ± s.d. values.

Having verified that TG2-179-1 inhibits BAP1, we investigated its impact on the viability of colon cancer cells. We treated the panel of colon cancer cell lines with increasing concentrations of TG2-179-1 and evaluated their viability by a colony formation assay. TG2-179-1 reduced the viability of all eight colon cancer cell lines in a dose-dependent manner regardless of their sensitivity to BAP1 depletion. The IC_50_ values of TG2-179-1 were less than 10 μM in all colon cancer cells, revealing no clear correlation with the requirement of BAP1 for survival (Fig. 6A,B). The cytotoxic activity of TG2-179-1 against the colon cancer cells was confirmed by an assay that measures short-term cell viability (Supplementary Fig. S3). Moreover, the Annexin-V assay showed that TG2-179-1 treatment increased apoptosis in HCT116 and SW48 cells in a dose-dependent manner (Fig. 6C,D). Notably, TG2-179-1 also killed CCD-18Co and CCD-112CoN normal colon cells, which express BAP1 at very low levels, with cytotoxic activity comparable to that of colon cancer cells (Fig. 6E).

To gain insights into the level of stability of TG2-179-1 activity in cells, we treated HCT116 and SW48 cells with a suboptimal concentration of TG2-179-1 either in a single dose or in multiple doses with washout and compared the effects of these different treatment modes on cell viability by a colony formation assay (Fig. 6F). A single treatment with TG2-179-1 at a given dose reduced cell viability by approximately 20%, whereas multiple treatments with the same dose led to a reduction of approximately 80% (Fig. 6G). These results, showing that the multiple replenishment mode is much more effective than a single treatment, suggest that TG2-179-1 is not stable enough to retain its cytotoxic activity for an extended period of time.

### TG2-179-1 exerts cytotoxic activity against colon cancer cells by targeting BAP1

**Figure 7:**
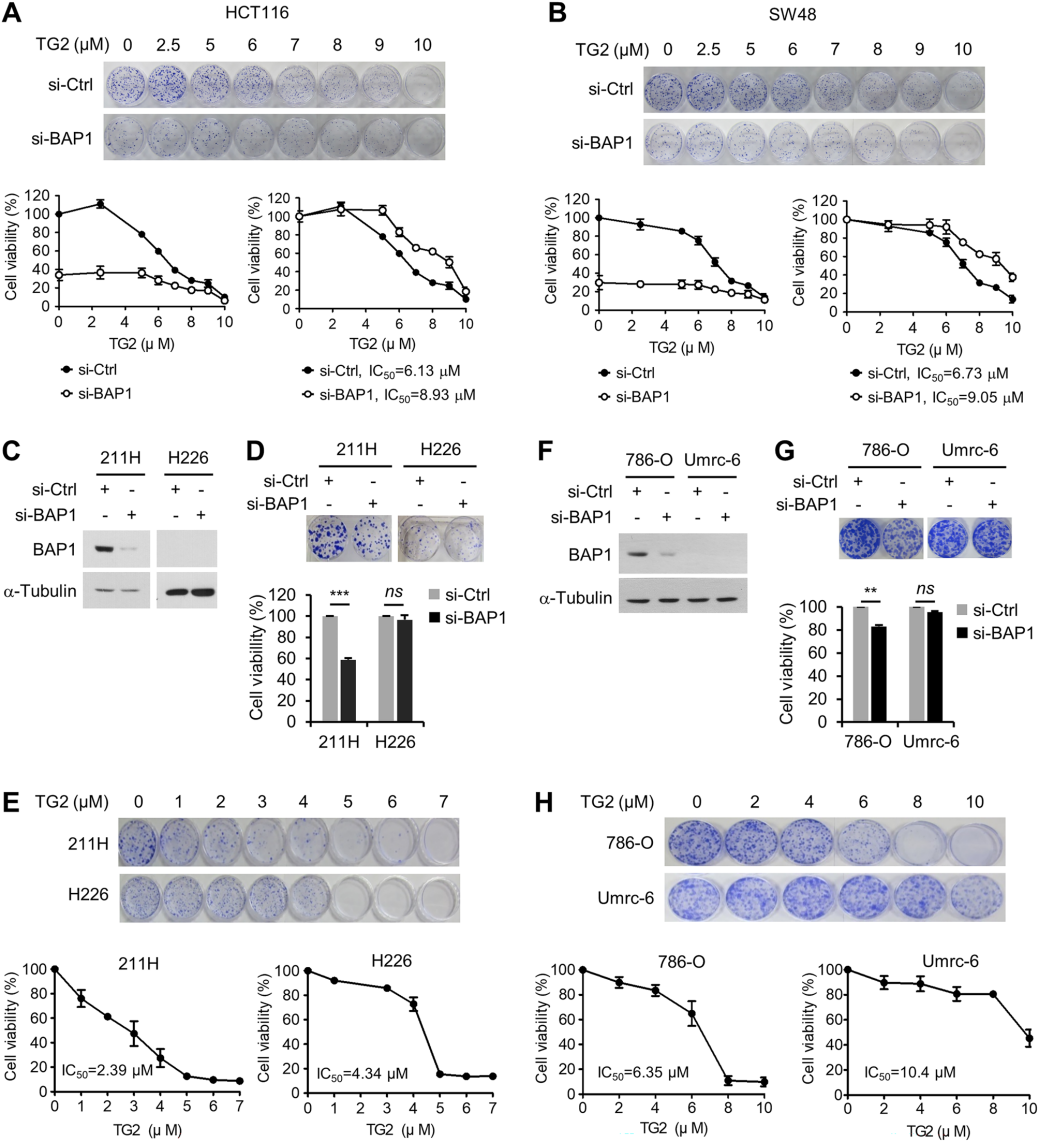
TG2-179-1 exerts cytotoxic activity against colon cancer cells by targeting BAP1. (**A**,**B**) After transfection with control or BAP1 siRNAs, HCT116 (**A**) and SW48 cells (**B**) were treated with increasing concentrations of TG2-179-1, and viability was evaluated by a colony formation assay. Representative results of the colony formation assay are shown (top). Relative viability was depicted graphically by setting the value for DMSO-treated (0 μM) si-Ctrl cells as 100 (lower left) or by setting each of the DMSO-treated si-Ctrl and si-BAP1 cell groups as 100 (lower right). The IC_50_ values are shown. (**C**) After transfection of MSTO-211H and H226 mesothelioma cells with control or BAP1 siRNAs, BAP1 expression was analyzed by immunoblotting. (**D**) The viability of the cells transfected as described in (**C**) was evaluated by a colony formation assay. Representative results are shown, along with a summary graph. *n* = 3; error bars, mean ± s.d. values. (**E**) MSTO-211H and H226 cells were treated with increasing concentrations of TG2-179-1, and viability was evaluated by a colony formation assay. Representative results are shown, along with a summary graph. *n* = 3; error bars, mean ± s.d. values. The IC_50_ values are shown. (**F**–**H**) Results of similar experiments as in C-D with 786-O and Umrc-6 ccRCC cells. (**F**) Immunoblots for BAP1 depletion. (**G**) Results of the colony formation assay after BAP1 depletion. *n* = 3; error bars, mean ± s.d. values. (**H**) Results of the colony formation assay after TG2-179-1 treatment. *n* = 3; error bars, mean ± s.d. The IC_50_ values are shown.

The observation that TG2-179-1 still killed BAP1-insensitive colon cancer cells (HCT15 and LoVo) raised the question of whether TG2-179-1 exerts its cytotoxic activity by targeting BAP1. We treated HCT116 cells with increasing concentrations of TG2-179-1 after BAP1 depletion and evaluated cell viability by a colony formation assay. BAP1 depletion greatly reduced cell viability, as observed previously (Fig. 7A). Notably, the sensitivity of the cells to TG2-179-1 treatment decreased after BAP1 depletion, as determined by the increased IC_50_ value (Fig. 7A). In similar experiments with SW48 cells, BAP1 depletion rendered the cells less sensitive to TG2-179-1 (Fig. 7B). To confirm these results in cells of other types of cancer, we compared TG2-179-1 sensitivity between MSTO-211H and H226 mesothelioma cells, with BAP1 proficiency and deficiency, respectively. BAP1 depletion significantly reduced the viability of MSTO-211H cells, with no effect on H226 cells, as expected (Fig. 7C,D), showing that BAP1 is important for the viability of this cell line. Notably, TG2-179-1 exhibited cytotoxic activity against both cell lines, with a lower IC_50_ value in MSTO-211H cells (2.39 μM) than in H226 cells (4.34 μM) (Fig. 7E), indicating that BAP1-proficient cells are more sensitive to TG2-179-1 than BAP1-deficient cells. When we repeated similar experiments with a pair of BAP1-proficient (786-O) and BAP1-deficient clear cell renal cell carcinoma (ccRCC) cells (Umrc-6), we obtained virtually the same results. BAP1 depletion reduced the viability of 786-O cells and had no effect on Umrc-6 cells (Fig. 7F,G), and TG2-179-1 killed both cell lines, with stronger cytotoxicity against 786-O cells than against Umrc-6 cells (IC_50_, 6.35 versus 10.4 μM) (Fig. 7H). These results suggest that TG2-179-1 exerts its cytotoxic activity against colon cancer cells as well as other types of cancer cells, such as mesothelioma and ccRCC, by targeting BAP1. However, TG2-179-1 kills both BAP1-insensitive and BAP1-depleted colon cancer cells as well as BAP1-null mesothelioma and ccRCC cells, indicating that TG2-179-1 targets BAP1 as well as other cellular proteins.

### TG2-179-1 induces replication defects in colon cancer cells and inhibits colon tumor growth

**Figure 8:**
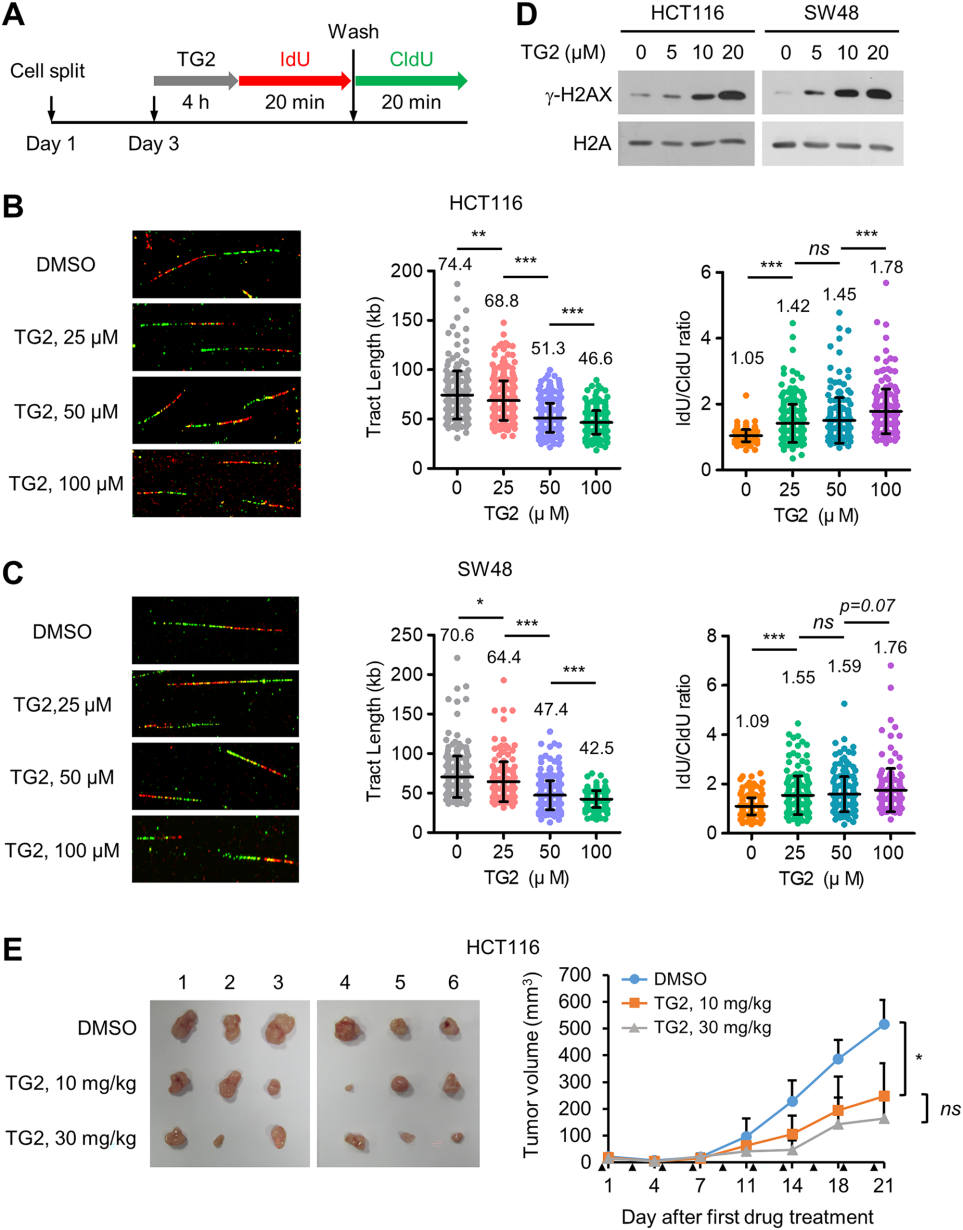
TG2-179-1 induces replication defects in colon cancer cells and inhibits colon tumor growth. (**A**) Experimental scheme. (**B**,**C**) HCT116 (**B**) and SW48 cells (**C**) were treated with increasing concentrations of TG2-179-1 for 6 h and were then subjected to a DNA fiber assay. (Left) Representative images of DNA fibers. (Middle) Summary of the IdU and CldU track lengths. Fiber counts for the scatter plot (in the same order as presented in the graph): 210, 304, 321, and 285 for HCT116; 251, 166, 285, and 201 for SW48. (Right) Summary of the IdU/CldU ratio. Fiber counts for the scatter plot (in the same order as presented in the graph): 162, 243, 181, and 213 for HCT116; 229, 174, 188, and 151 for SW48. (**D**) After cells were treated with increasing concentrations of TG2-179-1 for 48 h, histone extracts were prepared for immunoblotting. Representative data from three similar results are shown. (**E**) Results of the xenograft experiment showing that TG2-179-1 inhibits the tumorigenic growth of HCT116 cells. (Left) Images of tumors excised from mice. (Right) Summary graph. The arrowhead indicates the time point for drug injection. *n* = 6; error bars, mean ± s.d. values.

Next, we investigated the impacts of TG2-179-1 on DNA replication in colon cancer cells and tumor growth. We treated HCT116 cells with increasing concentrations of TG2-179-1 (up to 100 μM) and determined the impact of TG2-179-1 on replication by a DNA fiber assay as conducted previously (Fig. 8A). TG2-179-1 treatment reduced the speed of replication fork progression in a dose-dependent manner, as evidenced by the decreased IdU/CldU track lengths (Fig. 8B, left and middle panels). TG2-179-1 treatment also significantly increased the average IdU/CldU track length ratio compared with the value of 1 in untreated cells (Fig. 8B, left and right panels), indicating that TG2-179-1 induced fork stalling and collapse. Consistent with these results, TG2-179-1 treatment increased the level of γ-H2AX in a dose-dependent manner (Fig. 8D). Similar experiments with SW48 cells showed virtually the same results (Fig. 8C,D). These data show that TG2 treatment, similar to BAP1 depletion, induces defective fork progression and replication stress in colon cancer cells.

Finally, we determined whether TG2-179-1 inhibits the growth of colon tumors by a xenograft experiment as described above. We transplanted HCT116 cells into athymic nude mice and allowed the cells to grow for 7 days to form tumors of a certain size. TG2-179-1 was then administered to mice via intraperitoneal injection, and the tumor volume was measured every 3–4 days until day 21. In addition, DMSO was injected as the vehicle control. Tumor growth was significantly inhibited in TG2-179-1-injected mice compared to control mice. While the tumor volume in control mice continued to increase until day 21 by approximately 23-fold, the volume of tumors in mice administered TG2-179-1 increased by approximately 12-fold (Fig. 8E). We observed no significant adverse events, including body weight (Supplementary Fig. S4), for the mice administered TG2-179-1 compared to the mice injected with DMSO. At two specific doses tested, 10 and 30 mg/kg, TG2-179-1 did not result in a significant difference in tumor inhibition activity (Fig. 8E). These results demonstrate the activity of TG2-179-1 in inhibiting the growth of colon tumors.

## Discussion

In this study, we showed that BAP1 is upregulated in colon cancer, with its expression significantly elevated in both cancer tissues and cancer cell lines compared to their normal counterparts. BAP1 depletion reduces colon cancer cell proliferation concomitant with increased apoptosis and defective DNA replication. BAP1 depletion inhibits the growth of colon tumors in xenograft mice. The BAP1 inhibitor TG2-179-1, which inhibits the DUB activity of BAP1 possibly by binding covalently to the active site, exhibits potent cytotoxic activity against colon cancer cells, with IC_50_ values of less than 10 μM, and inhibits the growth of colon tumors in xenograft mice. The data suggest that TG2-179-1 kills colon cancer cells by targeting BAP1, reproducing the results of BAP1 depletion, i.e., increased apoptosis and defective replication. This work therefore reveals that BAP1 acts oncogenically in colon cancer and is a potential therapeutic target for this cancer and suggests that TG2-179-1 can be developed as a therapeutic agent for colon cancer.

BAP1 functions as a tumor suppressor. Inactivating mutations and deletions of the BAP1 gene are found in multiple human cancers, such as mesothelioma, uveal melanoma and renal cell carcinoma, and BAP1 has many anticancer cellular functions, including suppression of cell proliferation and genome instability^3,32,35^. However, TCGA PanCancer Atlas analysis showed that somatic mutations and deep deletion of the BAP1 gene are rare in colorectal adenocarcinoma (10 of 594 cases, 1.68%). Our experimental data and database search results showed that BAP1 expression was greatly elevated in all analyzed colon cancer cell lines compared to normal colon cells and that BAP1 expression was upregulated at both the protein and mRNA levels in colorectal cancer tissues compared to normal colorectal tissues. Our data also showed that BAP1 depletion inhibited colon cancer cell proliferation and tumor growth. Consistent with our findings, immunohistochemical staining for BAP1 in a tissue microarray from a large and well-characterized cohort of colorectal cancer patients (2,751 tumor samples) showed that complete loss of BAP1 expression is extremely rare in colorectal tumors^46^. Collectively, these results strongly suggest that BAP1 plays a tumor-promoting role in colon cancer. A recent study showed that BAP1 is rarely mutated in breast cancers and promotes breast cancer cell proliferation and metastasis^34^. Our study therefore provides an additional example of the oncogenic role of BAP1 in common cancers.

The control of β-catenin stability by the Apc–Axin complex plays an important role in colon cancer cell proliferation. The ability of BAP1 to promote colon cancer cell proliferation, however, seems to be independent of this mechanism, since BAP1 depletion did not affect the levels of β-catenin and Axin. Our data instead showed that BAP1 depletion suppressed replication fork progression with concomitant induction of replication stress and apoptosis, suggesting that BAP1 promotes colon cancer cell proliferation by increasing the rate of DNA replication. A recent study showed that BAP1 forms a complex with the transcription factor KLF5 to stimulate cell cycle progression and promotes breast cancer cell proliferation by deubiquitinating and stabilizing KLF5^31^. Given that KLF5 plays an oncogenic role in colon cancer^47^, BAP1 may also contribute to colon cancer cell proliferation and tumor growth via KLF5 stabilization.

TG2-179-1 was identified based on its ability to inhibit the DUB activity of BAP1 as determined using the artificial substrate Ub-AMC. We verified the ability of TG2-179-1 to inhibit the DUB activity of BAP1 toward AMC-Ub as well as toward the physiological substrate H2A-Ub assembled into nucleosomes. Our in silico docking model predicted that TG2-179-1 binds to BAP1 with its fluorobenzene and thiolane rings deeply embedded into the active site pocket, which sterically allows TG2-179-1 to interact covalently with Cys91 in the active site via its electrophilic nitrile carbon atom. In support of this prediction, TG2-179-1 inhibited BAP1 even after immunoprecipitation of TG2-179-1-bound BAP1 with extensive washing. However, only partial inhibition was observed. We therefore propose that the covalent interaction between BAP1 and TG2-179-1 is reversible. Consistent with this hypothesis, TG2-179-1 exhibited much higher toxicity in cells after multiple replenishment compared to after a single treatment. Unfortunately, we failed to detect BAP1 peptides containing TG2-179-1–bound Cys91 by mass spectrometry due to the difficulty of peptidase digestion of the region containing Cys91.

TG2-179-1 exhibits potent cytotoxic activity against colon cancer cells, with IC_50_ values of < 10 μM. Our results suggest that TG2-179-1 exerts this activity by targeting intracellular BAP1. First, TG2-179-1 treatment produces the same effects as BAP1 depletion in colon cancer cells: reduced replication fork progression, increased fork stalling, and increased apoptosis. Second, BAP1 depletion renders colon cancer cells less sensitive to TG2-179-1. Third, BAP1-proficient mesothelioma and ccRCC cells are more sensitive to TG2-179-1 than their BAP1-deficient counterpart cells. Finally, TG2-179-1 treatment, similar to BAP1 depletion, inhibits colon tumor growth in mice. However, our data show that TG2-179-1 also kills BAP1-deficient mesothelioma and ccRCC cells with significantly strong cytotoxicity. In addition, TG2-179-1 still exhibits strong cytotoxicity against CCD-18Co and CCD-112CoN normal colon cells, which express BAP1 at very low levels. These results suggest that TG2-179-1 exerts its cytotoxic activity both by targeting BAP1 and via BAP1-independent mechanisms and that its activity is not cancer cell specific. TG2-179-1 might have off-targets that belong to the BAP1-independent pathways critical for cell survival and proliferation in general. Therefore, it will be of great interest to identify such off-targets because it may permit the use of molecular modeling approaches to design novel TG2-179-1-based drugs that specifically target BAP1. Identification of such off-targets might also provide insights into exploring potential clinical applications of TG2-179-1 in personalized treatment of colon cancer, for example, focusing on patients lacking activity of the off-targets. In this regard, our work provides an experimental rationale for developing TG2-179-1 as a potential therapeutic agent in colon cancer treatment.

## Supplementary Information

Supplementary Information.

## Data Availability

The detailed experimental procedures and the materials used in this study will be freely available upon request. Please contact jongkwon@ewha.ac.kr.
