# Supplementary material for: Targeting BAP1 with small compound inhibitor for colon cancer treatment
Source: Sci Rep. 2023 Feb 8;13:2264. doi: 10.1038/s41598-023-29017-w (PMC9908887; doi:10.1038/s41598-023-29017-w)

Supplementary Material

Targeting BAP1 with small compound inhibitor for colon cancer treatment

Minhwa Kang, Seul Gi Park, Shin-Ai Lee, Soyi Kim, Daye Lee, Mukesh Eknath Shirbhate, So-Yeon Youn, Kwan Mook Kim, Sun-Shin Cha, Jongbum Kwon

1. Supplementary Figures
2. Uncropped full-length original blots
3. Supplementary Methods

A

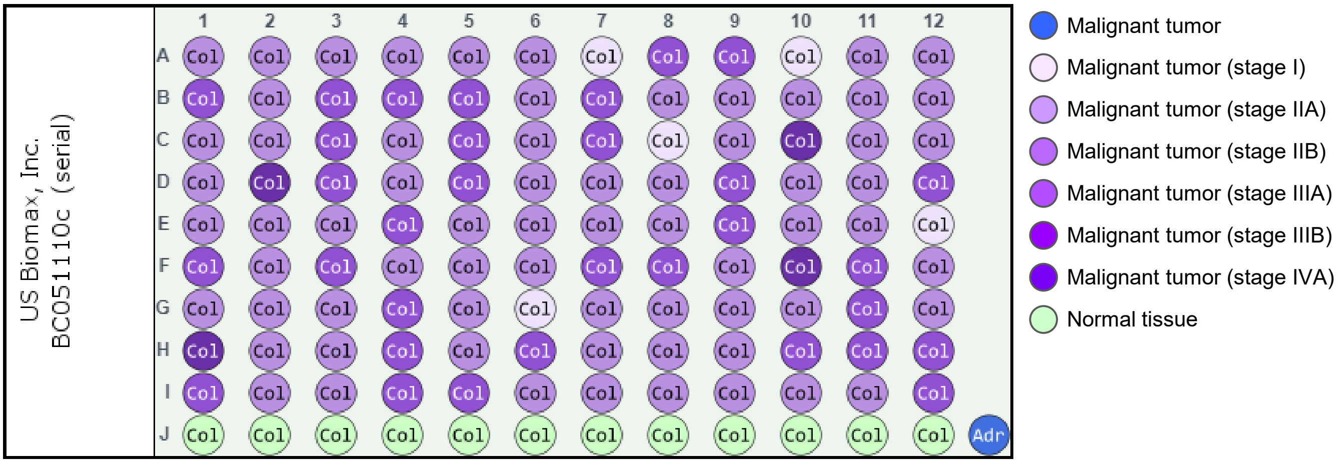

Microarray Panel	Colon adenocarcinoma and normal colon tissue microarray, containing 106 cases of colon adenocarcinoma, 2 cases of signet ring cell carcinoma, 12 normal colon tissue, single core per case
Cores	120
Cases	120
Row number	10
Column number	12
Core Diameter (mm)	1
Thickness (µm)	5
Tissue Array Type	FFPE
Species	Human

B

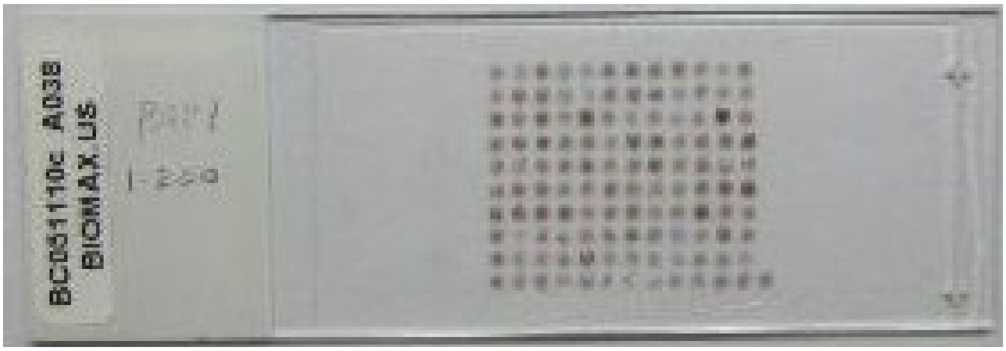

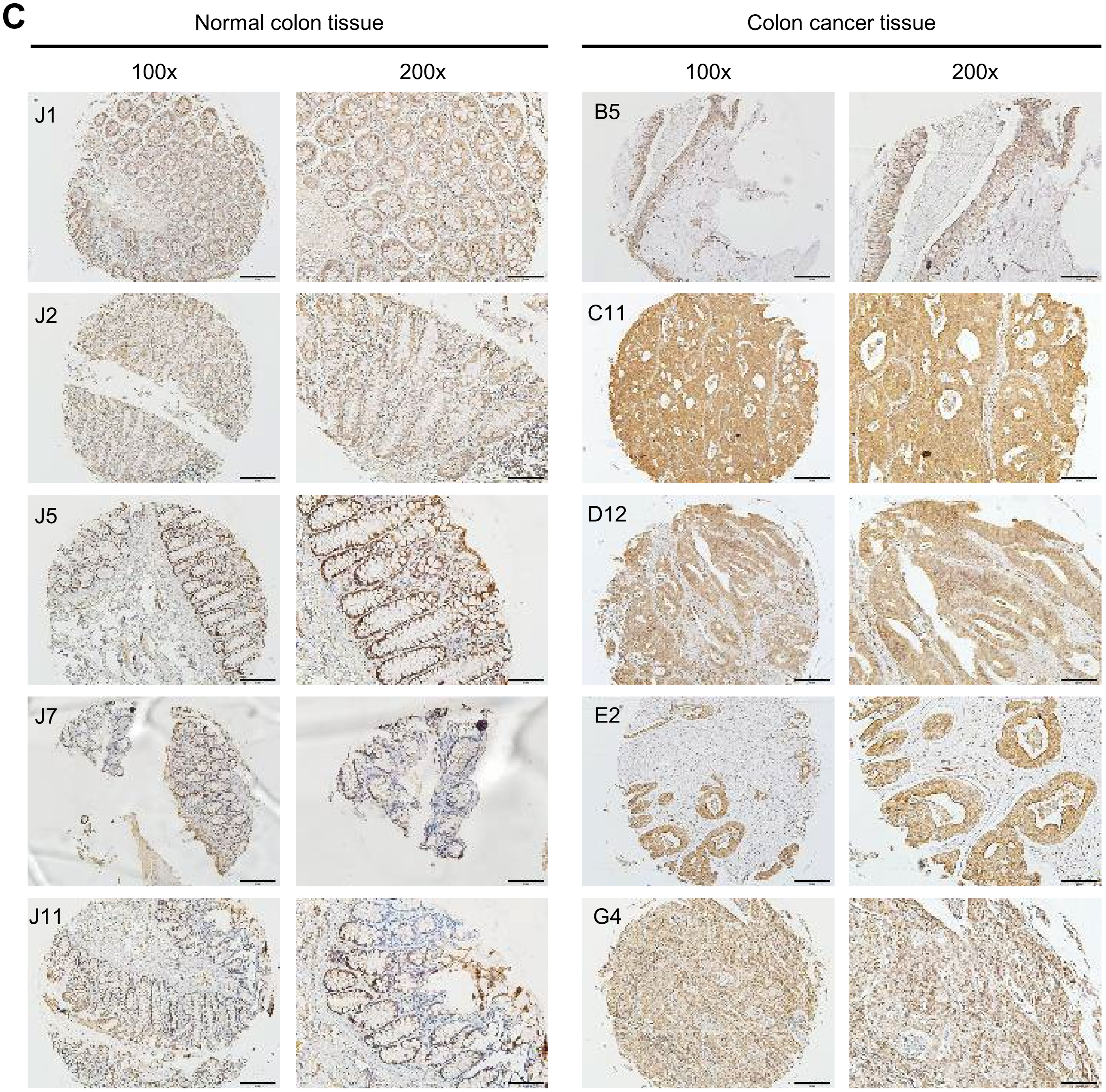

Supplementary Figure 1. Supplementary data for Figure 1D-F.

(A) Display of the BC051110c panel of colon carcinoma and normal tissue microarray. (B) The images of the whole microarray with BAP1 staining. (C) Additional images of immunohistochemical staining for BAP1.

	APC
HCT116	Wild type
HCT15	R2166 (nonsense), I1417fs (frameshift), R727M (missense), I1779M (missense)
HT29	E853(nonsense), T1556fs (Frame Shift)
SW48	Wild type
SW480	Q1338 (nonsense)
RKO	Wild type
DLD-1	Q1469L (missense), I1417fs (Frameshift)
LoVo	R1114(nonsense), R2816Q (missense), M1431fs (Frameshift)

Supplementary Figure 2. Supplementary data for Figure 4A and B.
The mutation status for the gene encoding Apc in various colon cancer cell lines.
(Reference: Cancer Research (2014) 74:3238-47. doi: 10.1158/0008-5472.CAN-14-0013)

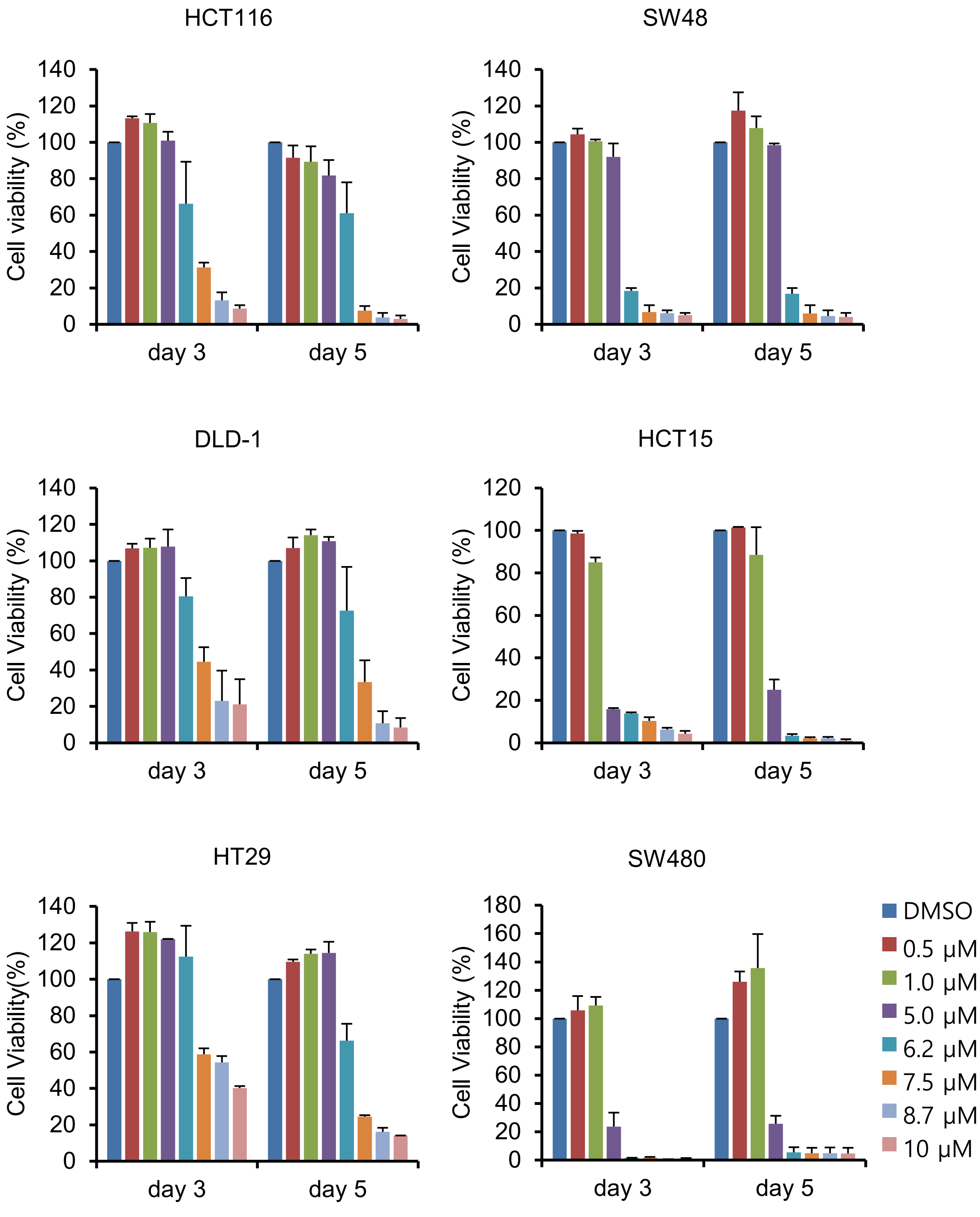

Supplementary Figure 3. Supplementary data for Figure 6A and B.

Colon cancer cells were incubated with increasing concentrations of TG2-179-1, and the short-term viability was evaluated at day 3 and 5 by an MTS assay. *n*=3; error bars, mean ± s.d. values.

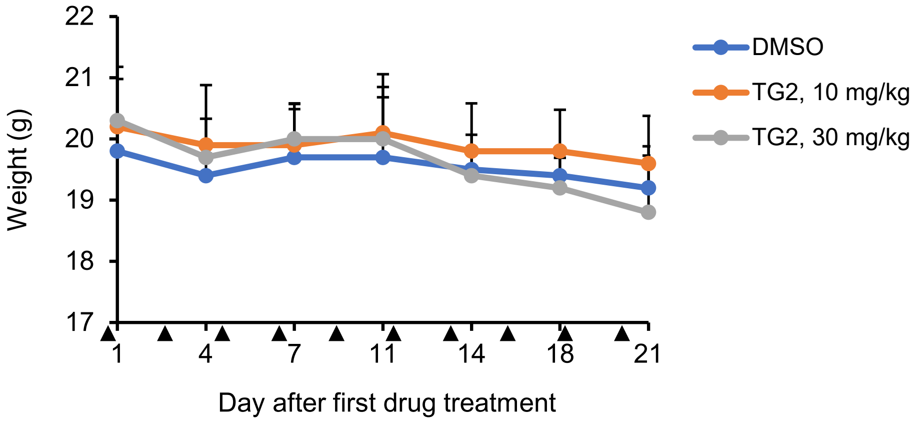

Supplementary Figure 4. Supplementary data for Figure 8E. Mouse body weight was determined at the same time as the measurement of tumor volume. Arrowhead indicates the time point for drug injection. *n*=3; error bars, mean ± s.d. values.

Uncropped full-length original blots

Fig 1C

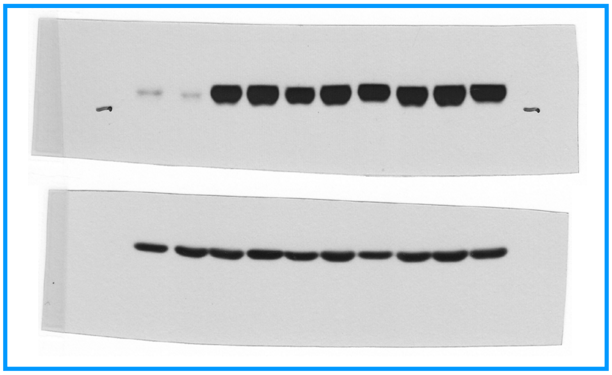

Fig 2C - HCT116

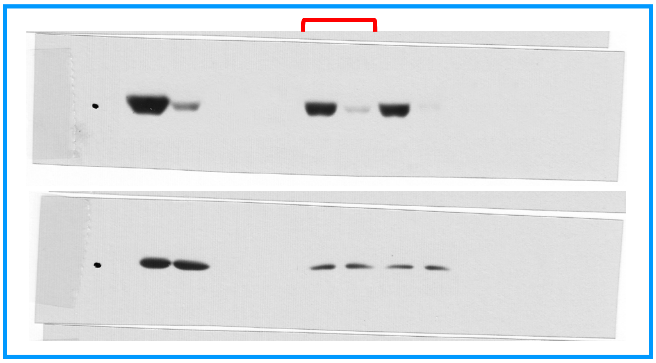

Fig 2C – HCT15

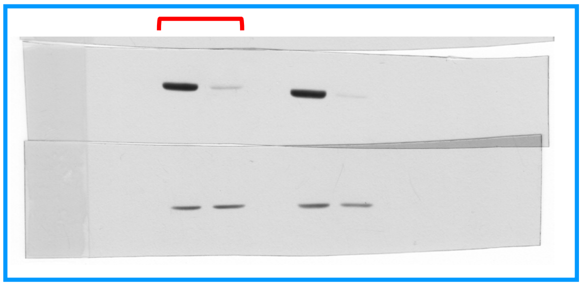

Fig 2C – HT29

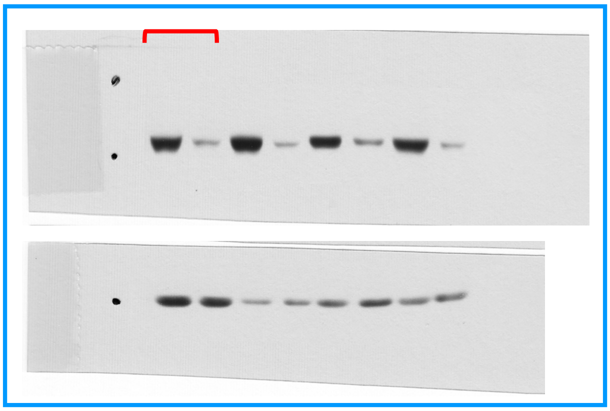

Fig 2C – SW48

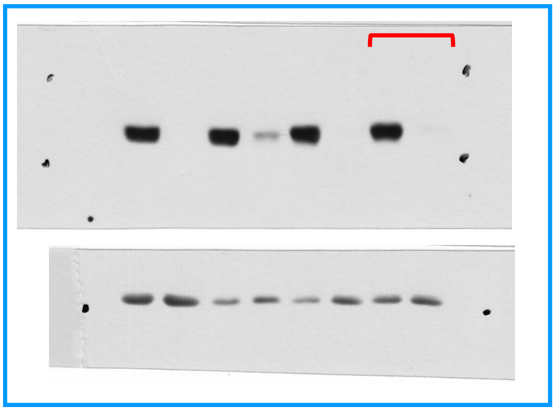

Fig 2C – SW480

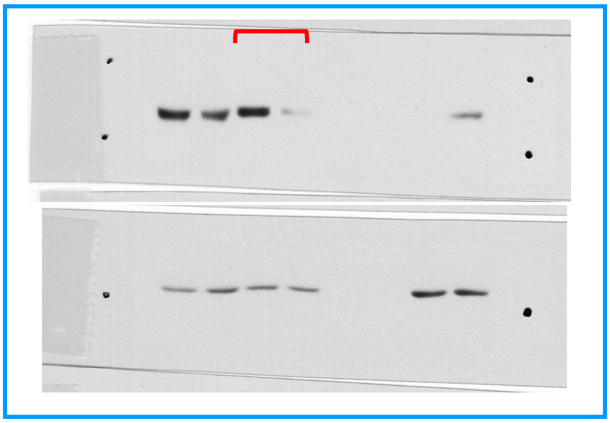

Fig 2C – RKO/LoVo

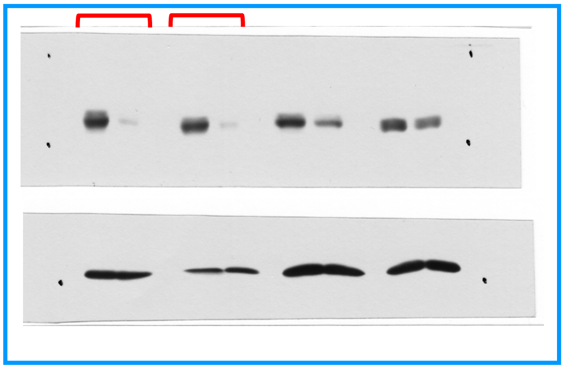

Fig 2C – DLD-1

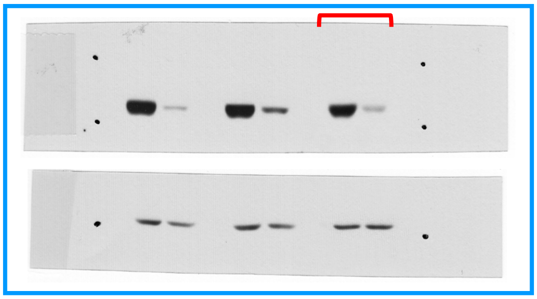

Fig 2D

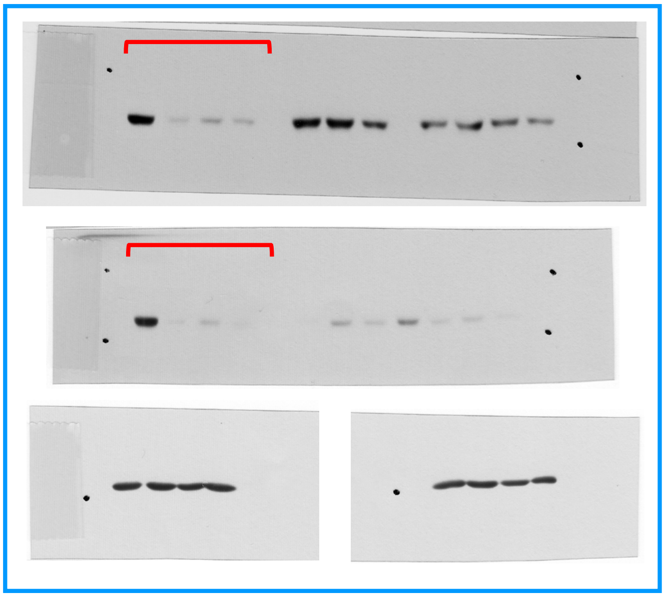

Fig 2E

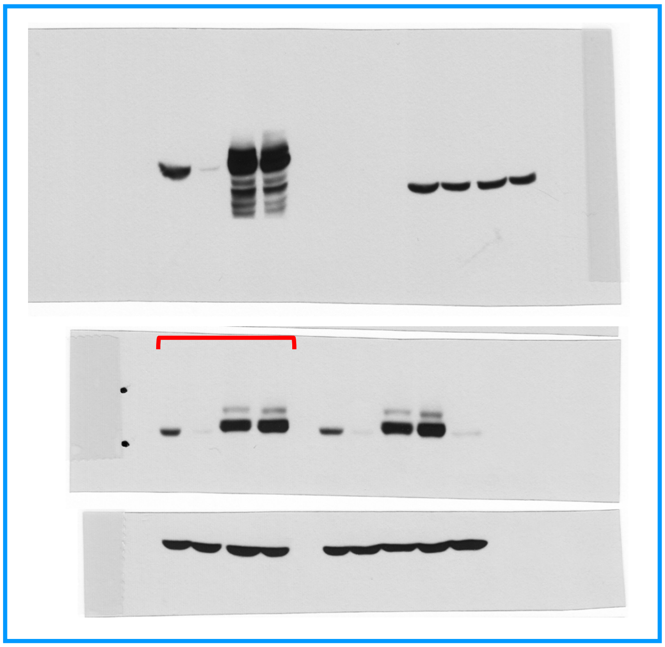

Fig 3

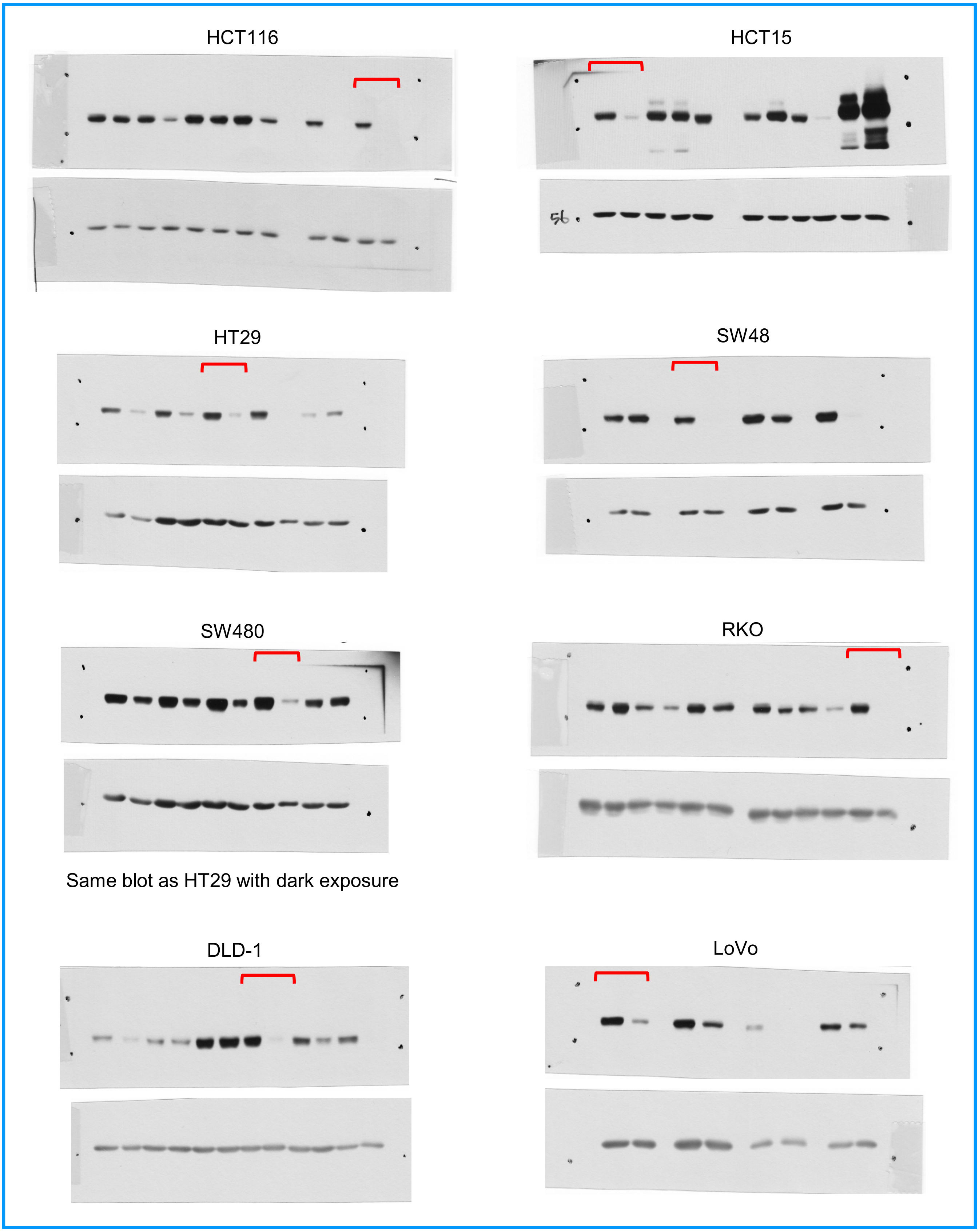
HCT116
HCT15
HT29
SW48
SW480
RKO
Same blot as HT29 with dark exposure
DLD-1
LoVo

Fig 4A

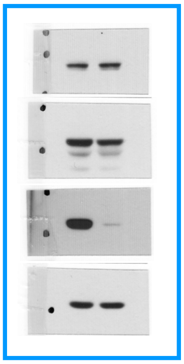

Fig 4B

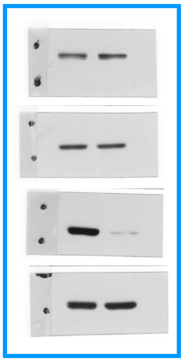

Fig 4D

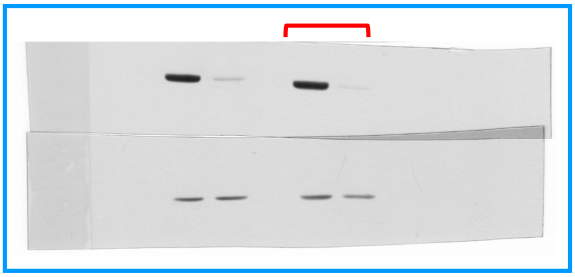

Fig 4F

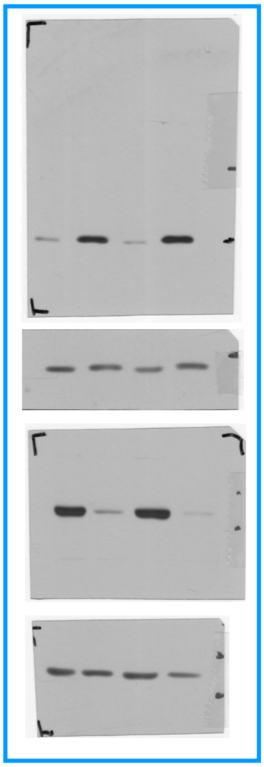

Fig 4E

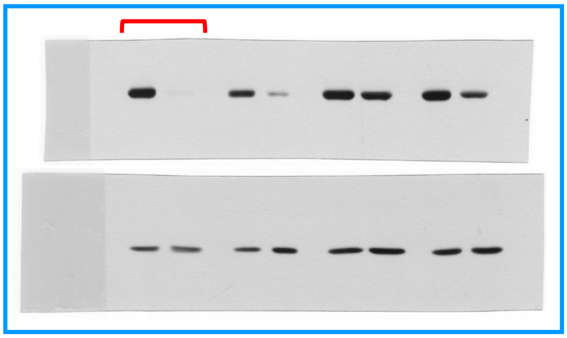

Fig 4G

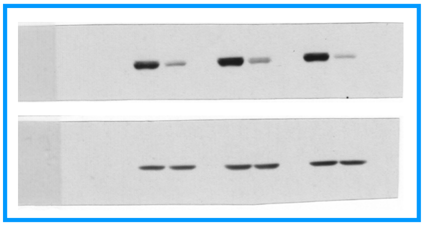

Fig 5D

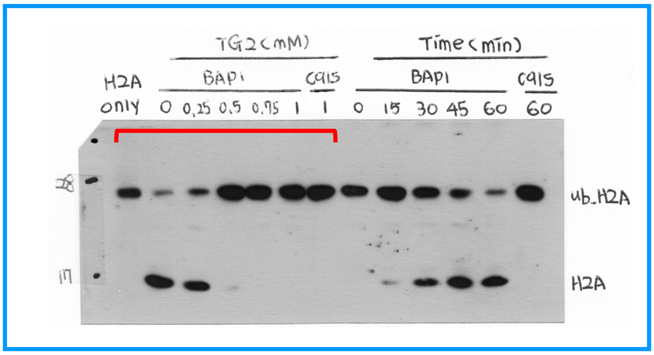

Fig 8D

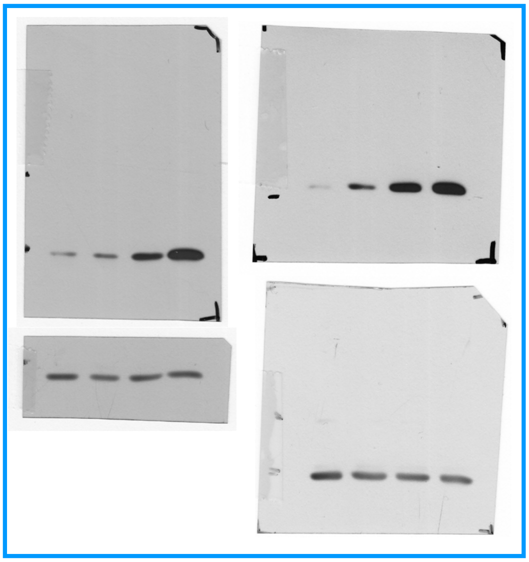

Fig 7C

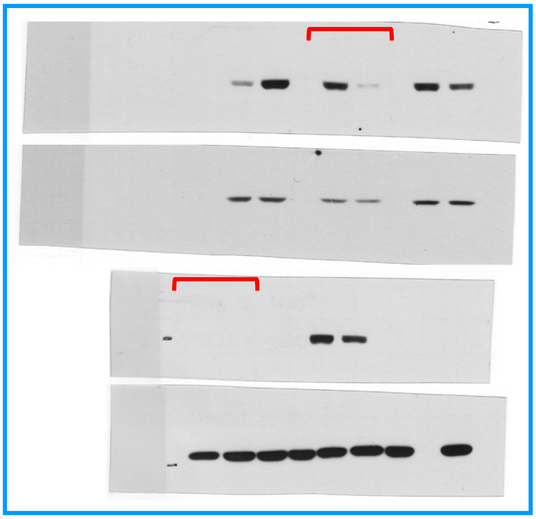

Fig 7F

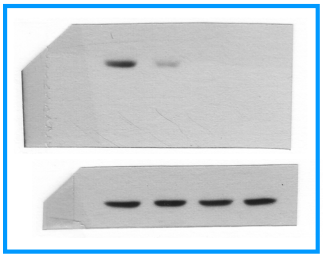

Supplementary Method

Synthesis of TG2-179-1

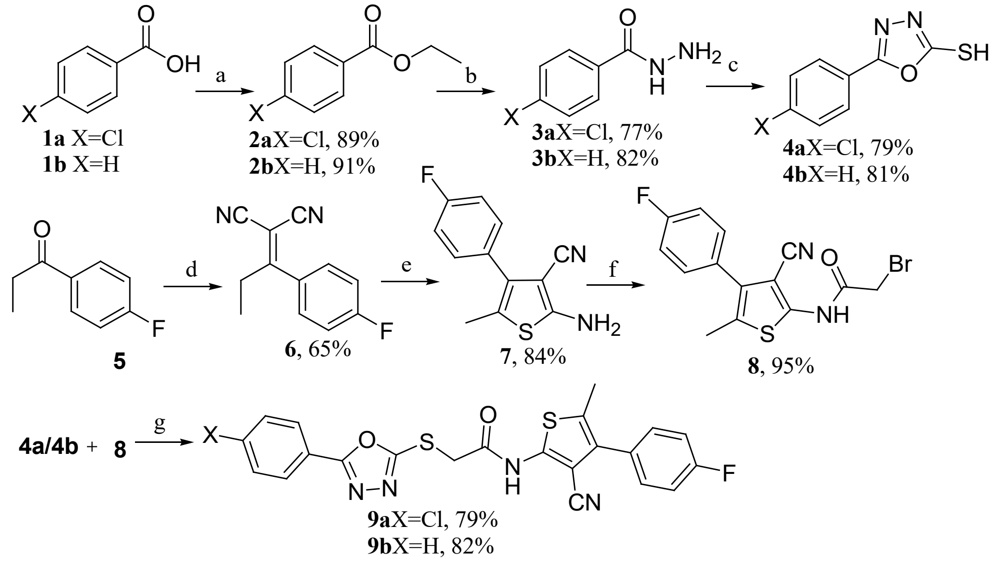

Reagents and conditions: (a) H_2SO_4, EtOH, 12 h, reflux; (b) $H_4N_2{\cdot}H_2O$, 85 °C 14 h; (c) CS_2, KOH, EtOH, 12 h, reflux, 1M HCl; (d) Malononitrile, CH_3CO_2H, ammonium acetate, toluene, 10 h, reflux; (e) Sulfur, Et_3N, EtOH, 5 h, reflux; (f) Bromoacetyl Bromide, Et_3N, CH_2Cl_2, 12 h, rt; (g) K_2CO_3, KI, CH_3CN, 6 h, rt.

Experimental details.

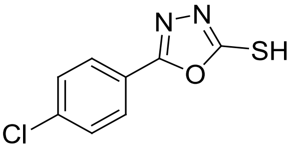

4a

To a solution of the hydrazide compound (**3**) (1.7 g, 10 mmol) in ethanol (15 mL) were added carbon disulphide (2 mL, 32.01 mmol) and potassium hydroxide (0.56 g, 10.69 mmol) at room temperature. The reaction mixture was reflux until the evolution of H_2S gas ceased (around 12 h). Excess solvents were evaporated under reduced pressure and the residue was dissolved in water and then acidified with dilute hydrochloric acid (1 M) to pH 5. The precipitate was filtered off, dried, and crystallized from ethanol give pure product 5-(4-chlorophenyl)-1,3,4-oxadiazole,2-thiol (**4a**) 1.7 g, solid, yield 79%, mp 158-160 °C. ^{1}H NMR (500 MHz, DMSO-*d6*): δ (ppm) 7.88-7.85 (m, 2H), 7.63 (t, J = 1.5 Hz, 2H), 3.49 (br s, 1H); ^{13}C NMR (DMSO-*d6*): δ (ppm) 178.185, 160.389, 160.363, 137.637, 130.295, 130.270, 128.579, 128.541, 122.078.

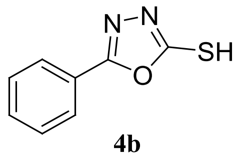

4b

Following the same procedure, (**4b**) was obtained yield 81%, mp 216-218 °C.
^{1}H NMR (500 MHz, DMSO-*d6*): δ (ppm) 7.87 (d, *J* = 7 Hz, 2H,), 7.57-7.65 (m, 3H), 3.34 (br s, 1H); ^{13}C NMR (DMSO-*d6*): δ (ppm) 178.177, 161.196, 132.962, 130.146, 126.766, 123.191.

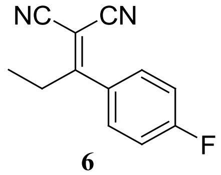

6

To the solution of 4- fluoro-propiophenone (**5**) (4.2g, 28 mmol) in 20 mL of toluene was added the malononitrile (2.1g, 32 mmol) at room temperature, and then ammonium acetate (500 mg, 6.5 mmol) and glacial acetic acid (2 mL) were added to the reaction mixture flask. The resulting mixture was refluxing vigorously, the water formed during reaction was removed by a Dean and Stark trap placed under the reflux condenser for 10 h. The solvent was evaporation and the left a residue was recrystallized from alcohol give pure product, 2-(1-fluoro-Phenylpropylidene)malononitrile (**6**) 3.6 g, Yield: 65%, solid, mp 74-78 °C. ^{1}H NMR (500 MHz, $CDCl_3$): δ (ppm) 7.51-7.54 (m, 2H), 7.21 (t, *J* = 9 Hz, 2H), 2.97 (q, *J* = 7 Hz, 2H), 1.12 (t, *J* = 10 Hz, 3H), ^{13}C NMR ($CDCl_3$): δ (ppm) 180.419, 165.986, 163.964, 130.851, 130.826, 130.270, 130.197, 84.625, 31.334, 13.053.

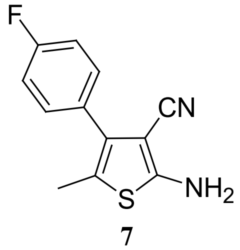

7

To a solution of 2-(1- fluoro-Phenylpropylidene)malononitrile (**6**) (2 g, 10 mmol) in ethanol (60 mL) was added the elemental sulfur of (0.35 g, 11 mmol) at room temperature, and then added a catalytic amount of piperidine into the reaction mixture. The reaction mixture was heated under reflux condenser for 5 hours, then allowed to cool to room temperature. The precipitated grayish yellow solid was filtered off and recrystallized from ethanol to give the 2-amino-4-(4-fluorophenyl)-5-methylthiophene-3-carbonitrile (**7**) 1.9 g, Yield: 84%, solid, mp 100-102 °C. ^{1}H NMR (500 MHz, $CDCl_3$): δ (ppm) 7.33 (d, *J* = 1 Hz, 2H), 7.12 (t, *J* = 1.2 Hz,

2H), 4.88 (s, 2H), 2.21 (s, 3H); ^{13}C NMR ($CDCl_3$): δ (ppm) 163.535, 161.565. 160.542, 134.253, 131.144, 131.081, 130.057, 130.032, 119.475, 116.244, 115.870, 115.700, 89.585, 13.499.

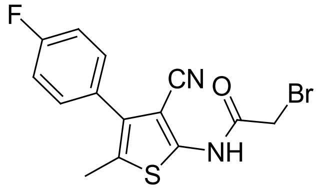

8

To a solution of 2-amino-4-(4-fluorophenyl)-5-methylthiophene-3-carbonitrile (**7**) (2.3 g, 10 mmol) in 30 mL CH_2Cl_2 was added the trimethylamine (1.5 mL, 11 mmol) at room temperature, and then added the Bromoacetyl Bromide of (2.1 g, 11 mmol) into the reaction mixture. The reaction mixture was keep at room temperature 12 h, column chromatography offered to give compound 2-bromo-N-(3-cyano-4-(4-fluorophenyl)-5-methylthiophen-2-yl)acetamide (**8**) 3.2 g, Yield 95%, solid, mp 110-112 °C. ^{1}H NMR (500 MHz, $CDCl_3$): δ (ppm) 9.51 (s, 1H) 7.35-7.38 (m, 2H), 7.18 (t, J = 2 Hz, 2H), 4.08 (s, 2H), 2.21 (s, 3H); ^{13}C NMR ($CDCl_3$): δ (ppm) 163.828, 163.310, 161.854. 146.219, 133.577, 131.280, 131.216, 129.055, 128.214, 116.150, 115.981, 114.592, 95.776, 27.580, 13.537.

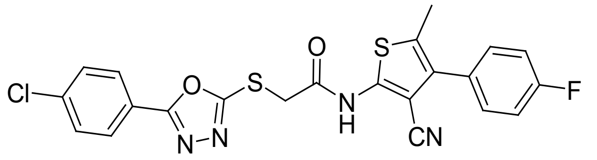

9a

To a solution of the 5-(4-chlorophenyl)-1,3,4-oxadiazole,2-thiol (**4a**) (1 g, 4.7 mmol) in CH_3CN (10 mL) were added the, K_2CO_3 (1.3 g, 9.4 mmol) and potassium Iodide (0.69 g, 0.41 mmol) at 0 °C and then added the 2-bromo-N-(3-cyano-4-(4-fluorophenyl)-5-methylthiophen-2-yl)acetamide (**8**) into the reaction mixture. The reaction mixture was keep at room temperature 6 h. Excess solvents were evaporated under reduced pressure and the residue was dissolved in water and then acidified with dilute hydrochloric acid (1 M). The precipitate was filtered off, dried, and crystallized from ethanol to give (**9a**) 1.8 g, yield 79%, mp 214-216 °C. ^{1}H NMR (500 MHz, DMSO-*d6*): δ (ppm) 12.14 (s, 1H) 7.95 (d, J = 14.5 Hz, 2H) 7.64 (d, J = 14.5 Hz, 2H), 7.41-7.46 (m, 2H), 7.33 (t, J = 9.5Hz, 3H), 4.52 (s, 2H), 2.25 (s, 3H); ^{13}C NMR (DMSO-*d6*): δ (ppm) 166.164, 165.098, 163.841, 163.323, 161.374. 146.794, 137.349, 133.394, 132.002, 131.935, 130.130, 129.775, 128.719, 127.404, 122.317, 116.174, 116.001, 115.012, 94.625, 35.842, 13.343; HRMS-(ESI+) *m/z* calcd. For $C_{22}H_{14}ClFN_4O_2S_2$ $[M + H]^+$, expected: 485.0288, found: 485.0290.

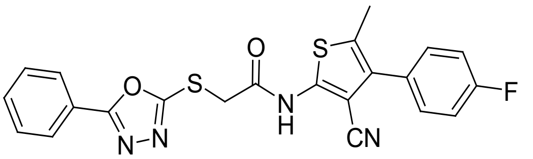

9b

Following the same procedure, (**9b)** was obtained yield 82%, mp 190-192 °C. ^{1}H NMR (500 MHz, $CDCl_3$): δ (ppm) 10.94 (s, 1H) 8.00 (d, *J* = 14.5Hz, 2H) 7.49-7.54 (m, 3H), 7.27-7.33 (m, 2H), 7.13 (t, *J* = 9Hz, 3H), 4.21 (s, 2H), 2.22 (s, 3H); ^{13}C NMR ($CDCl_3$): δ (ppm) 166.843, 164.959, 164.385, 163.718, 161.748. 146.321, 132.662, 132.312, 131.318, 131.250, 129.335, 129.259, 129.233, 127.828, 127.144, 123.318, 115.998, 115.828, 114.494, 95.057, 35.368, 13.512. HRMS-(ESI+) *m/z* calcd. For $C_{22}H_{15}FN_4O_2S_2$ $[M + H]^+$, expected: 451.0739, found: 451.0741.

^{1}H-NMR and ^{13}C-NMR spectra of representative compounds **4a** ^{1}H NMR (400 MHz, DMSO-*d6*); ^{13}C NMR (125 MHz, DMSO-*d6*)

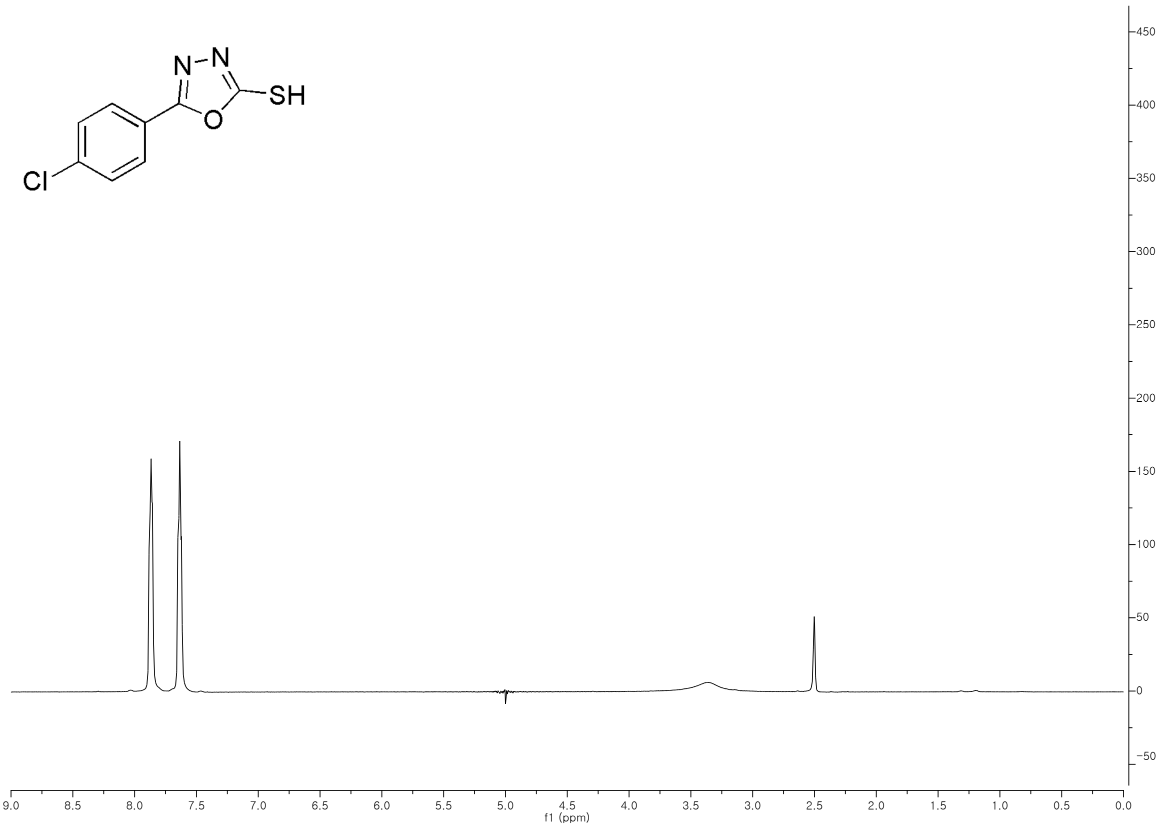

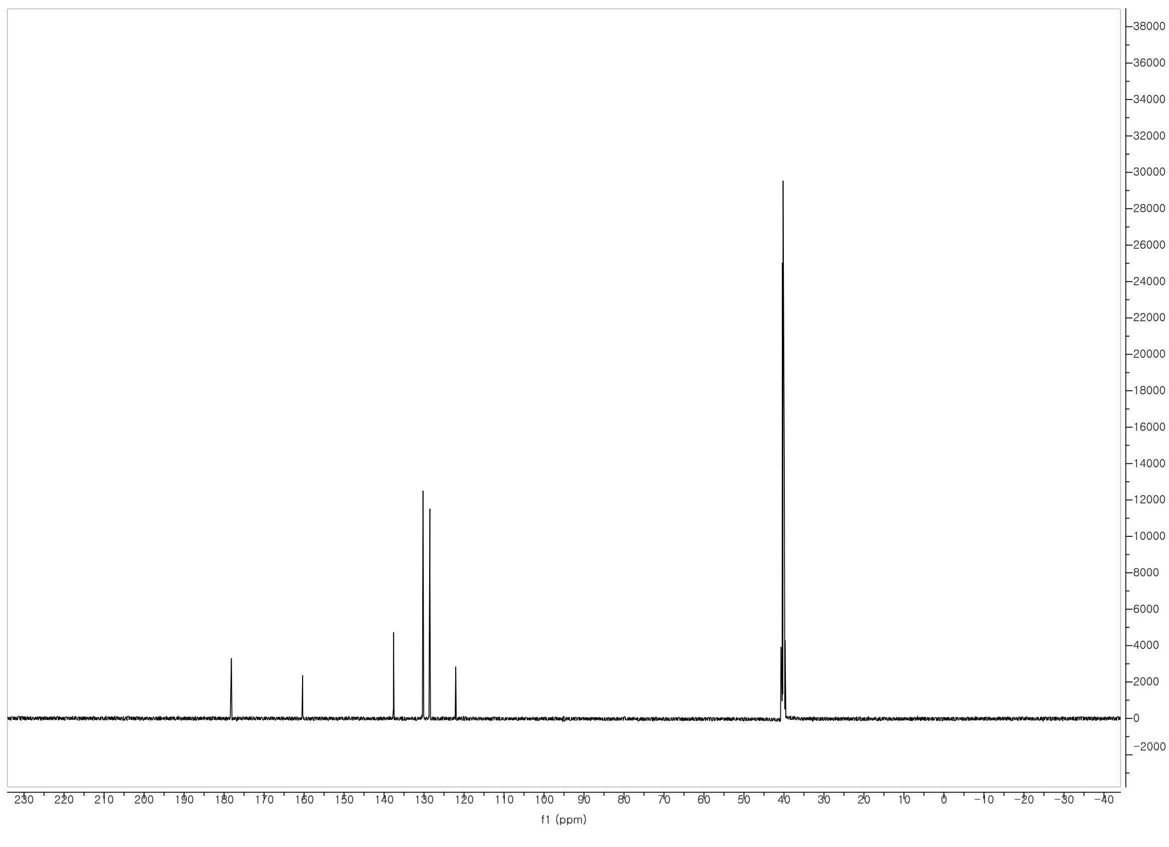

^{1}H-NMR and ^{13}C-NMR spectra of representative compounds **4b** ^{1}H NMR (400 MHz, DMSO-*d6*); ^{13}C NMR (125 MHz, DMSO-*d6*)

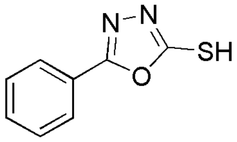

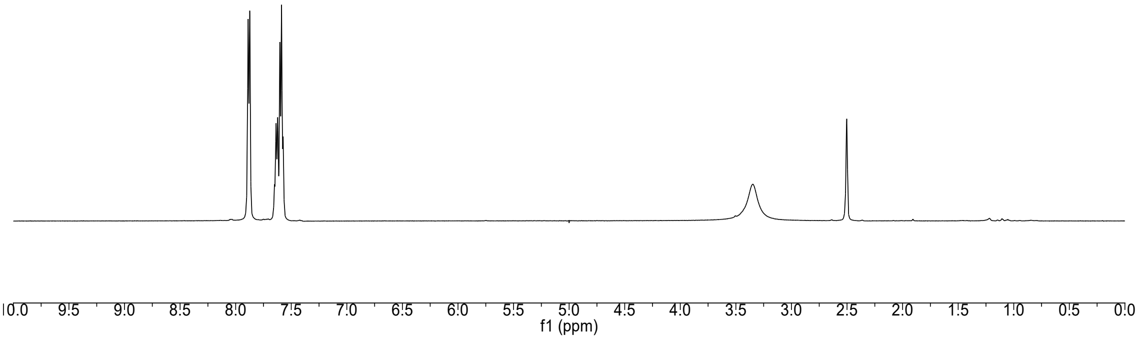

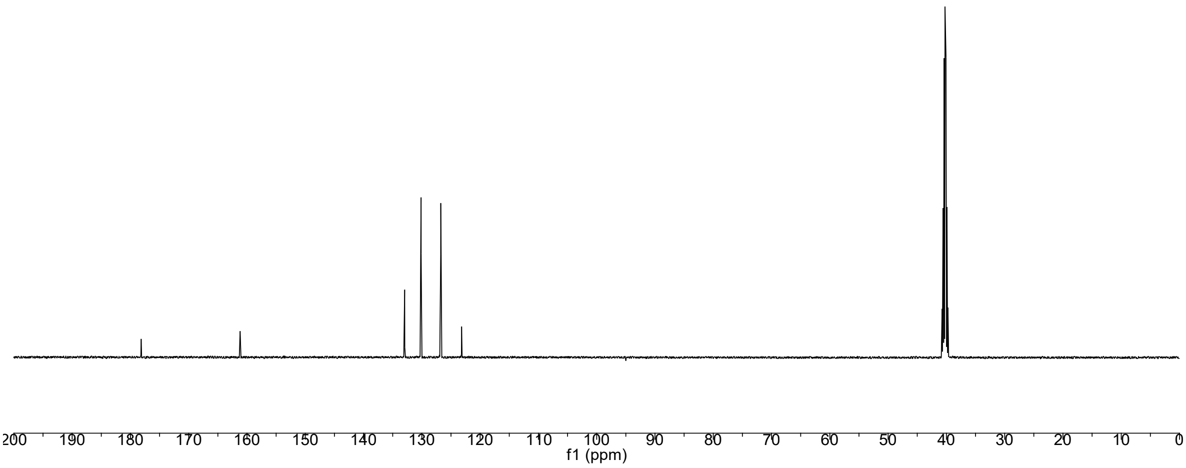

^{1}H-NMR and ^{13}C-NMR spectra of representative compounds **6** ^{1}H NMR (400 MHz, $CDCl_3$); ^{13}C NMR (125 MHz, $CDCl_3$)

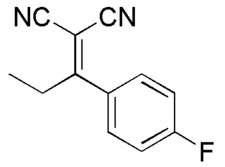

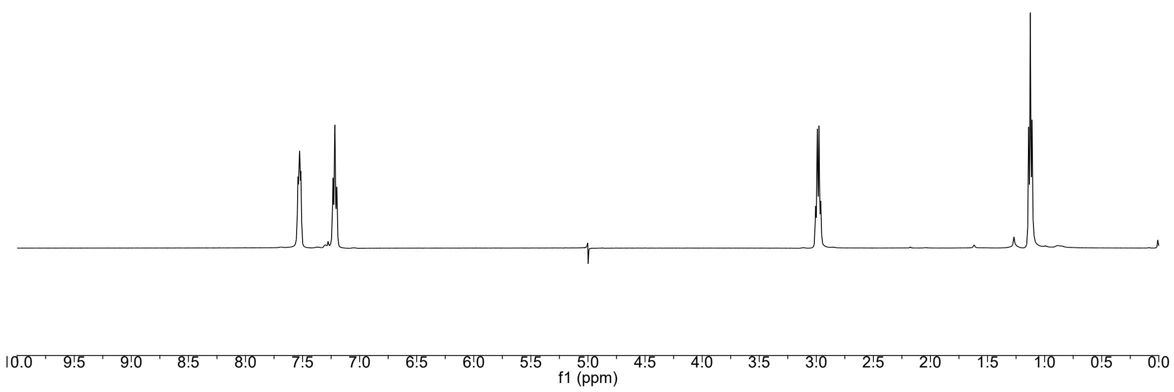

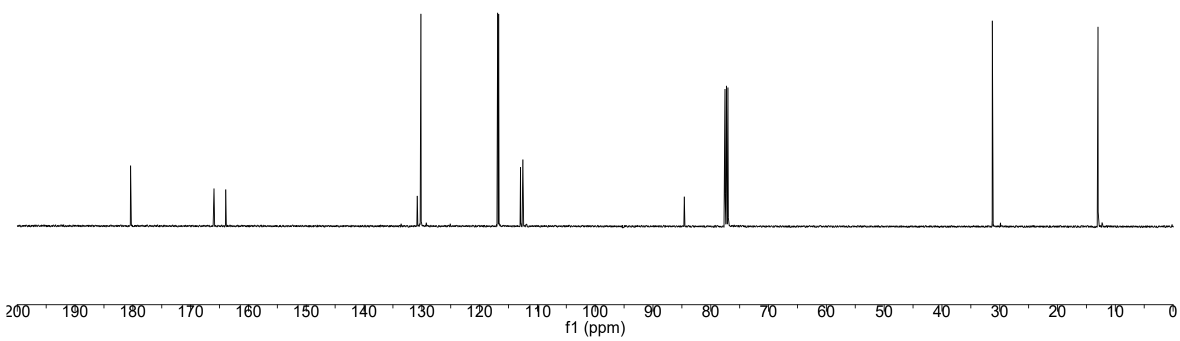

^{1}H-NMR and ^{13}C-NMR spectra of representative compounds **7** ^{1}H NMR (400 MHz, $CDCl_3$); ^{13}C NMR (125 MHz, $CDCl_3$)

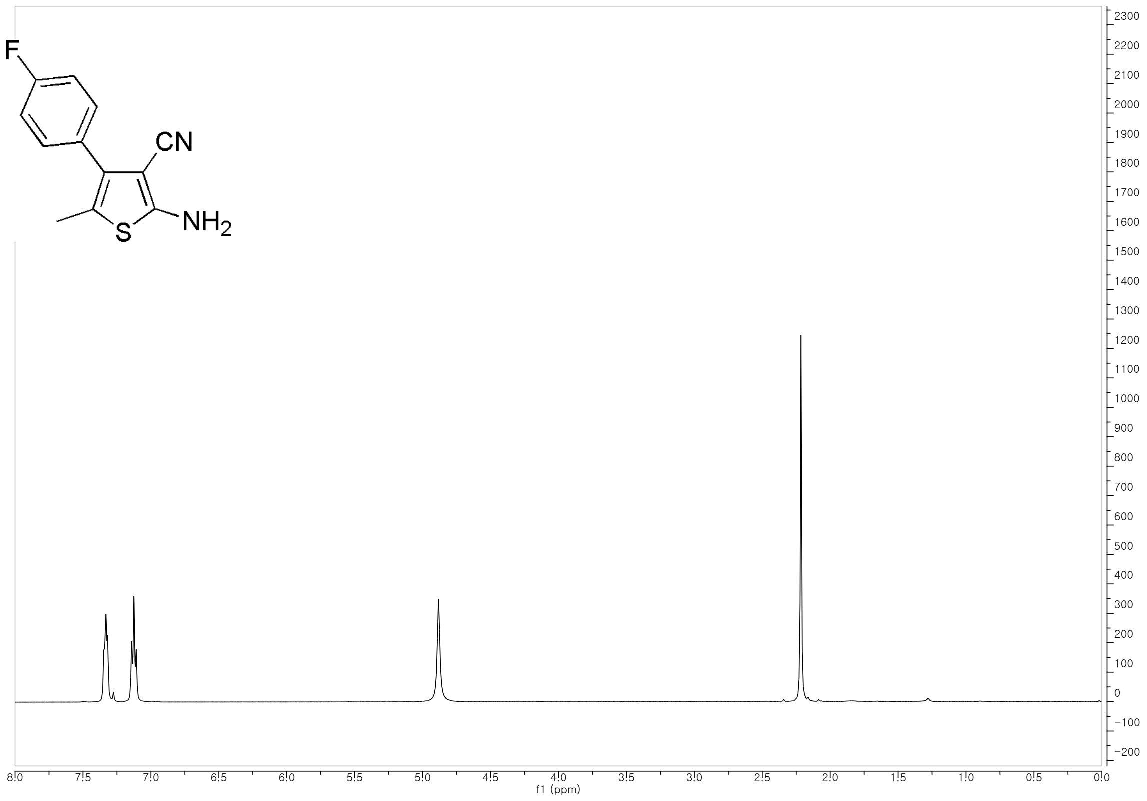

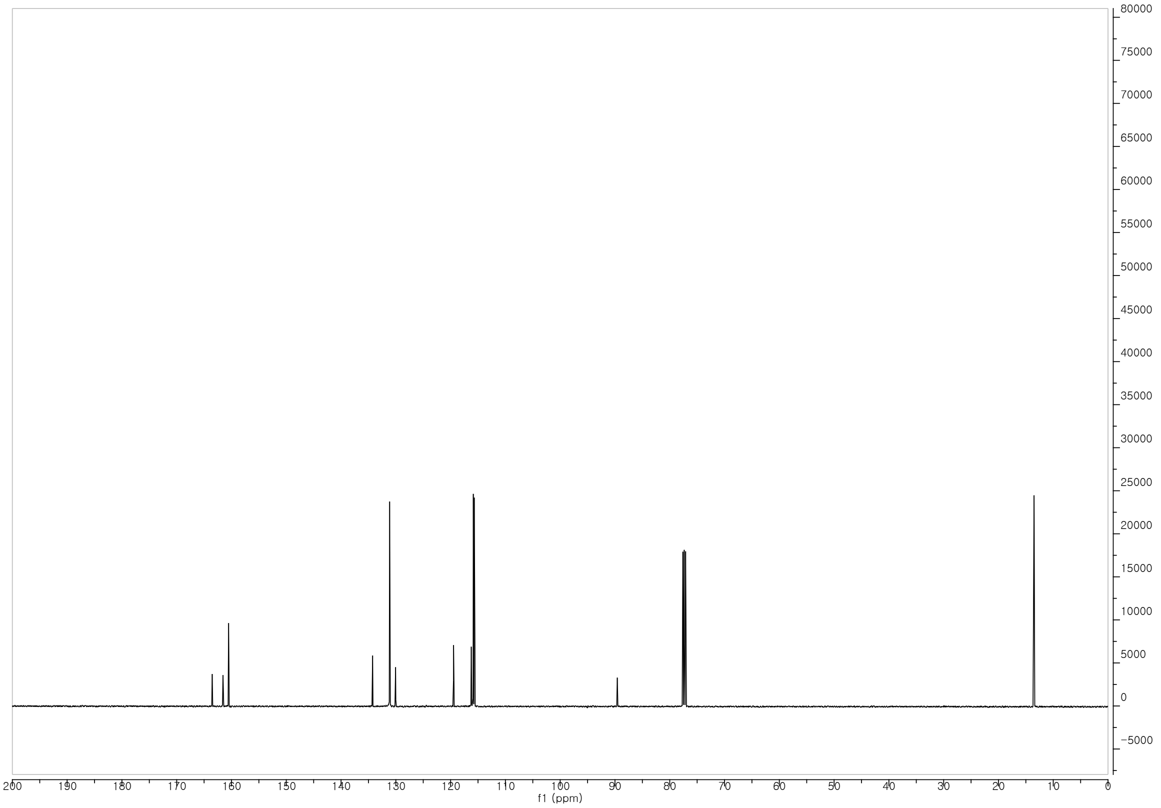

^{1}H-NMR and ^{13}C-NMR spectra of representative compounds **8** 1H NMR (400 MHz, $CDCl_3$); ^{13}C NMR (125 MHz, $CDCl_3$)

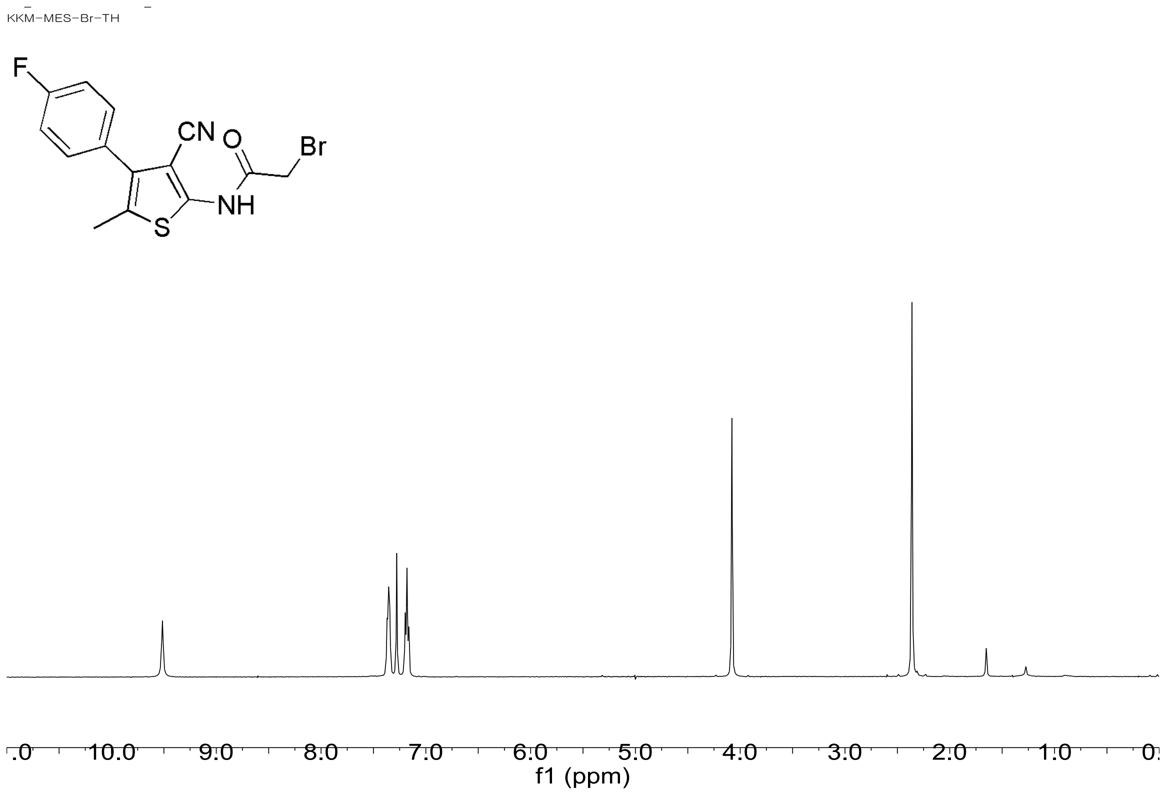

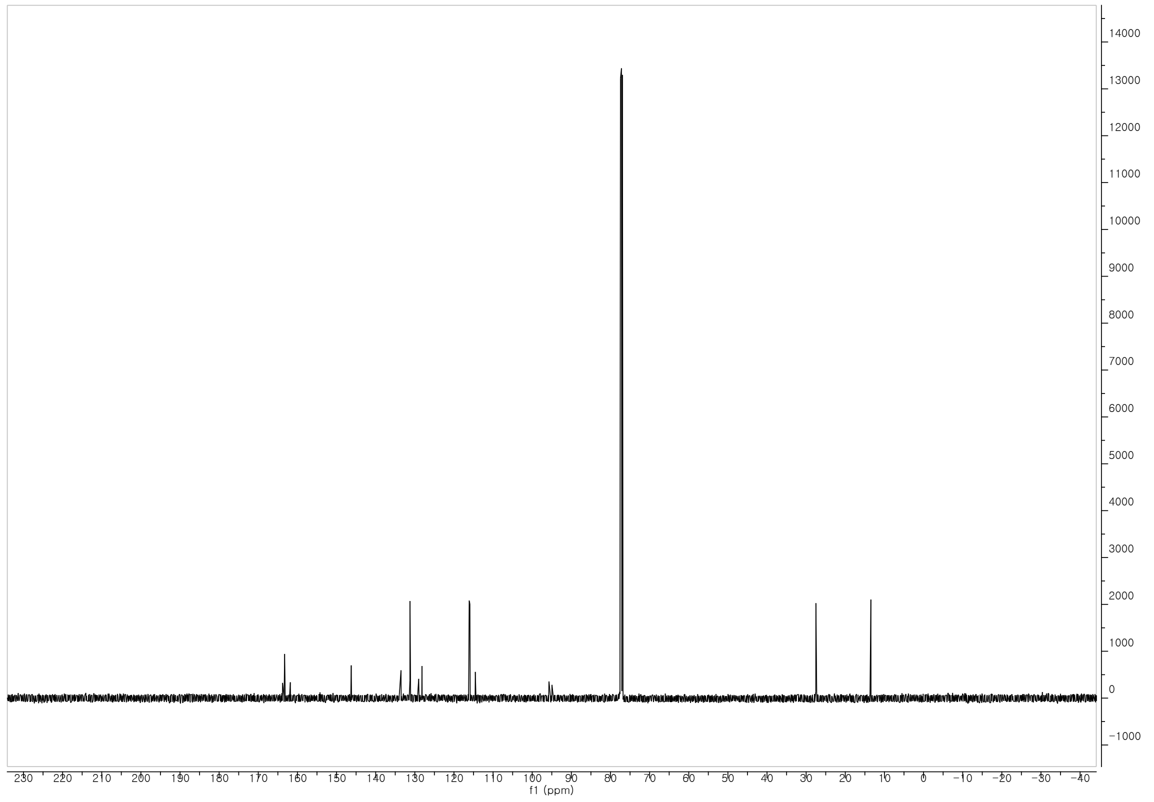

^{1}H-NMR and ^{13}C-NMR spectra of representative compounds **9a** ^{1}H NMR (400 MHz, DMSO-*d6*); ^{13}C NMR (125 MHz, DMSO-*d6*)

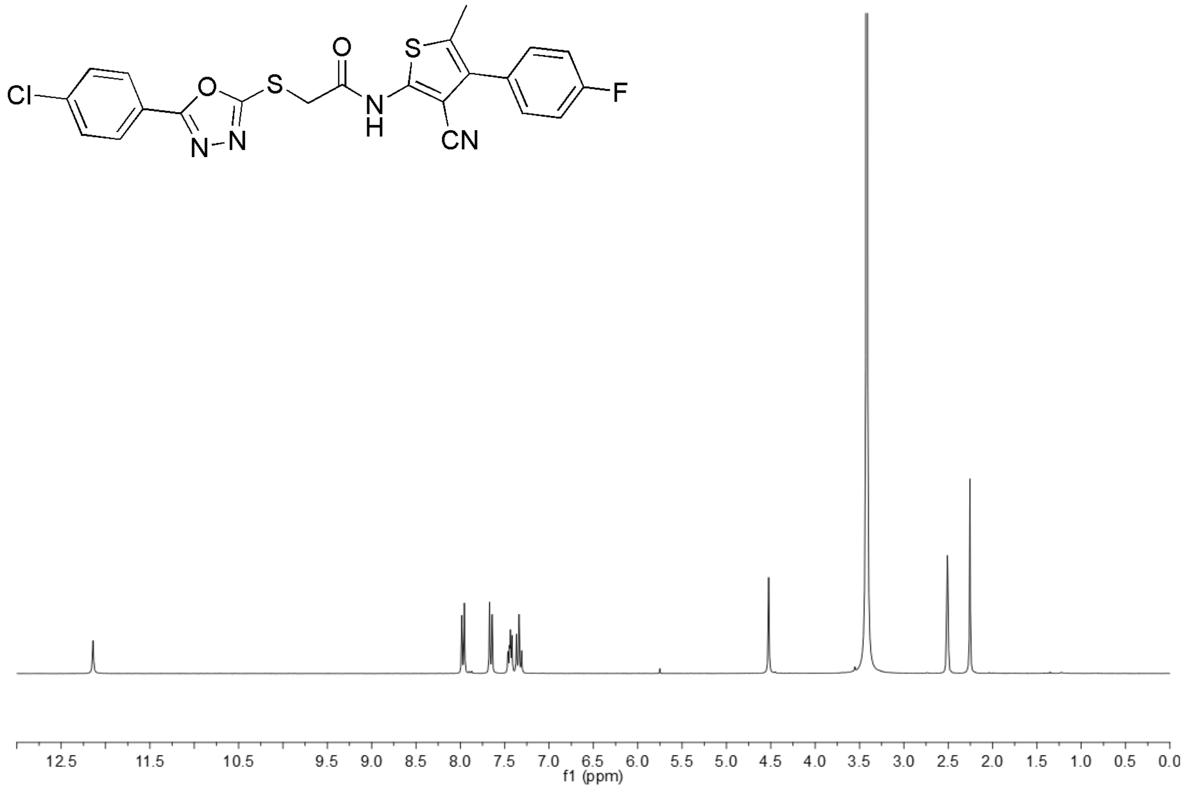

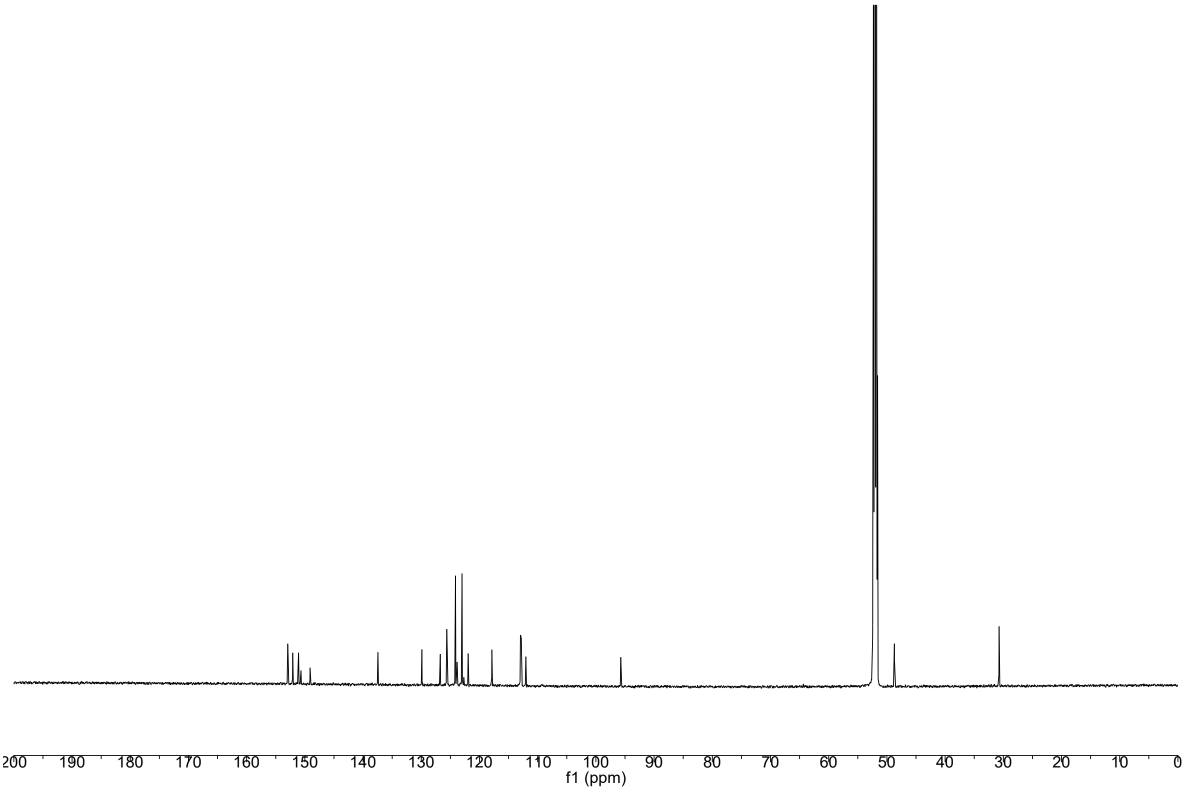

^{1}H-NMR and ^{13}C-NMR spectra of representative compounds **9b** ^{1}H NMR (400 MHz, $CDCl_3$); ^{13}C NMR (125 MHz, $CDCl_3$)

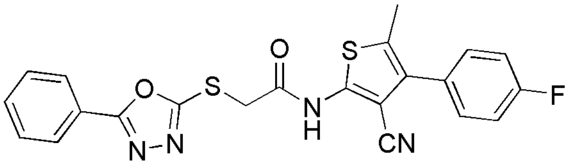

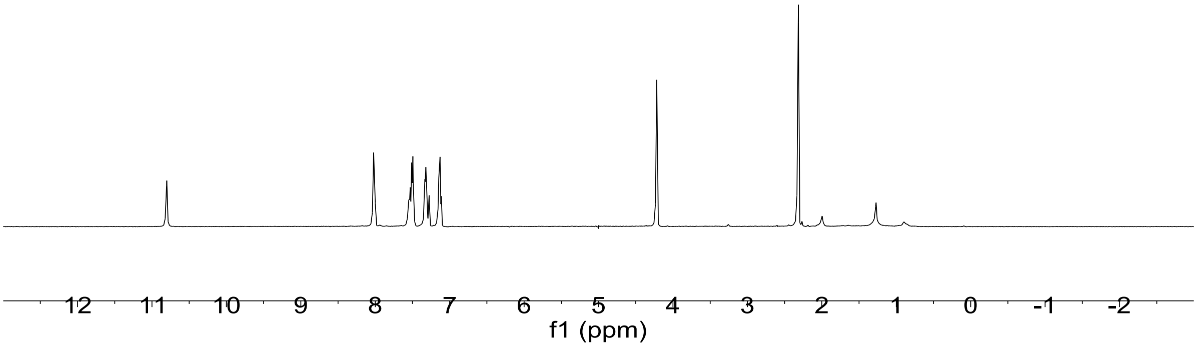

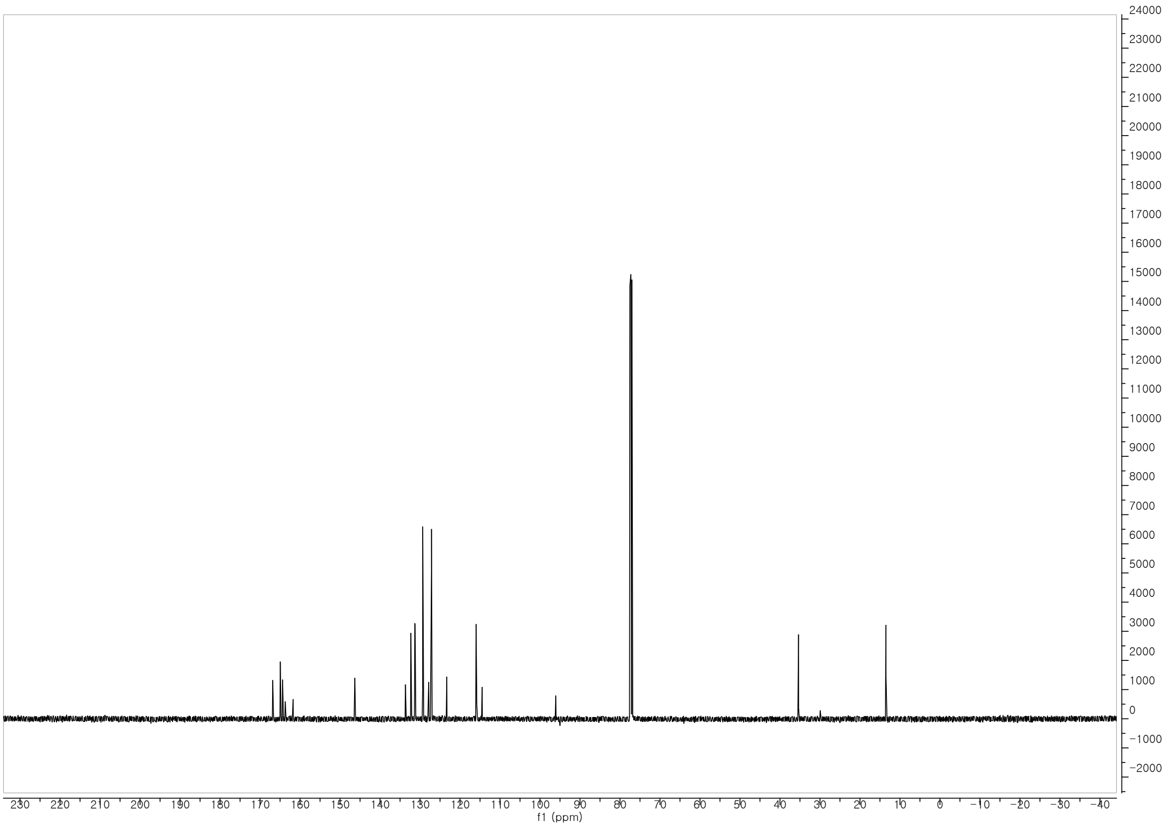

Supplement: Supplementary file 1 — Supplementary Information. [file 41598_2023_29017_MOESM1_ESM.pdf]
